# Specific and broad-spectrum antibacterial effectors of type VI secretion system drive competition of *Stenotrophomonas rhizophila* against bacteria from seed microbiota

**DOI:** 10.1128/spectrum.03532-25

**Published:** 2026-06-15

**Authors:** Boris Taillefer, Yassine Dairy, Agathe Brault, Martial Briand, Pierre Charpin, Jean Armengaud, Alain Sarniguet

**Affiliations:** 1 Univ Angers, Institut Agro, INRAE, IRHS, SFR QUASAV, Angers, France; 2 Département Médicaments et Technologies pour la Santé, Université Paris-Saclay, CEA, INRAE 27048, Bagnols-sur- Cèze, France; Illinois State University, Normal, Illinois, USA

**Keywords:** secretion system, T6SS, effector, toxin-antitoxin, regulation, interbacterial competition, seed microbiota

## Abstract

The type VI secretion system (T6SS) is a bacterial weapon that injects deadly effectors (T6Es) directly into a target competitor encountered in a microbial community. *Stenotrophomonas rhizophila* CFBP13503 (*Sr*), a seed-borne bacterium, uses its T6SS to target a wide range of other bacterial species, impacting seed microbiota assembly, and controlling phytopathogen transmission from seed to seedling. How the large T6E repertoire and other features contribute to *S. rhizophila*’s impact was investigated. Using transcriptional and translational reporters combined with fluorescence microscopy and proteomic approaches, we confirmed the expression and production of nine effectors secreted by a highly dynamic T6SS. This resulted in killing target cells through four major cell death phenotypes, with different frequencies depending on the target species. The systematic deletion of single effectors revealed a general synergy in killing a wide range and diverse seed-borne bacteria, but also a target-specific killing. Comparative genomics suggests that *Sr*-T6Es are commonly shared in the *Stenotrophomonas* genus diversity but are also highly plastic and acquired from diverse bacterial families. Inversely, T6SS-resistance markers, such as capsule and orthologous immunities, are weakly distributed in seed microbiota, and do not explain resistance to *Sr*-T6SS. Importantly, two amidases explained the major T6SS antibacterial activity, and only the Rhs-fused effector targeted a Gram-positive bacterium. Our work depicts the generalist or specific role of each effector in the *S. rhizophila* T6SS activity targeting either aggressive, closely related, broad, or Gram-positive bacteria from seed microbiota.

**IMPORTANCE:**

The type VI secretion system (T6SS) is largely distributed among seed-borne bacteria. However, it remains important to understand how this interbacterial competition weapon could provide a competitive advantage for some strains and influence microbiome assembly during seed-to-seedling transmission. The *Stenotrophomonas rhizophila* CFBP 13503 strain, a seed-associated bacterium, cumulated several T6SS features like a constitutive and highly dynamic T6SS associated with 12 putative antibacterial T6Es that shape microbiota assembly through broad and strain-specific targeting within seed microbiota. Hence, genomic analysis suggests that *S. rhizophila* T6E repertoire is adaptive thanks to genetic gain and loss or through gene regulation. In the context of microbiome engineering, the relevance of this study is to highlight the need to associate broad-effective and competitive *S. rhizophila* strains, as well as T6SS-resistant strains, in bacterial synthetic communities as seed inoculum against phytopathogens.

## INTRODUCTION

Microorganisms associated with plant seeds, referred to as the seed microbiota, play an important role in plant development through mutualistic or pathogenic interactions (1). Dormant seed is a harsh environment where bacteria must compete for limited nutrients and space. Then, seed germination and seedling emergence offer plenty of resources that lead to intensive interbacterial competition (2, 3). To efficiently compete within the seed microbiota, microorganisms can engage the type VI secretion system (T6SS) that injects toxic effectors (T6E) directly into a target cell, leading to growth inhibition or cell death (4). Alternatively, T6SS can be used to secrete effectors for nutrient acquisition or oxidative stress resistance (5). T6SSs are encoded in genetic clusters and are found in 25% of Gram-negative bacteria (6). T6SS genetic clusters or dedicated effector modules are widely exchanged through horizontal gene transfers and play a role in bacterial adaptation (6, 7). For instance, at least one T6SS cluster is found in up to 60% of seed- and plant-associated bacteria (8, 9). Despite its large distribution and important role in shaping microbial communities, few studies have attempted to explain the relative role of the T6SS of microbiota members (9, 10).

The T6SS is composed of 14 core components and other accessory proteins involved in the assembly. Specifically, it is assembled through three subcellular complexes (11). The membrane complex (TssJLM) anchors the baseplate (TssEFGK) equipped with the effector-loaded spike (VgrG-PAAR), which serves as a docking hub for the tail polymerization (Hcp tube and TssBC sheath) coordinated by TssA. Once assembled, the T6SS can contract to propel the effector in the milieu or directly into a target cell. The contracted TssBC sheath is then disassembled by the dedicated ClpV ATPase to build a new system (11).

VgrG is often capped with a metal-bound PAAR. T6Es are loaded either on the VgrG spike or into the Hcp tube, with or without the help of an adaptor protein Tap, or encapsulated in an Rhs cage. They can also be genetically encoded as a VgrG, Hcp, Rhs, or PAAR-fused effectors (12). T6Es can target all essential cellular processes and components through dedicated phospholipase, amidase, NADase, protease, RNase, and DNase activities (12). Antibacterial T6Es are always accompanied by a cognate immunity (T6I) that allosterically blocks the toxic activity, thus preventing self and sibling intoxication.

Target strains can also resist T6SS attacks through orthologous immunity (13, 14) or immunity-independent defense mechanisms such as capsule (15), EPS production (16, 17), fimbriae (18), biochemical modification of the molecular target, or by a global cellular defense program (19–21). Dead cell debris was also reported to lower the T6SS potency by creating a physical barrier between the aggressor and the target (22). However, aggressive strains have evolved to alleviate this constraint by mostly using lytic effectors, such as amidases or phospholipases (23). Since the T6SS function and specific effector-immunity pairs act as a strong selective pressure on both attacker and target strains, they naturally evolve rapidly through gene exchange, gain and loss, duplication, or diversification events (24–27). Several studies reported large T6E-I repertoires composed of more than one effector and poly-immunity clusters (27–31), though their number and ecological role are weakly investigated. It has been proposed that a large repertoire not only improves the target range but is also a bet-hedging strategy, minimizing T6SS resistance of target strains and increasing T6SS potency in changing environments (30).

*Stenotrophomonas rhizophila* CFBP13503 (*Sr*) is a seed-associated, plant growth-promoting bacterium of the *Lysobacteriaceae* family (32) that uses a T6SS to increase its fitness by competing with residents of the seed microbiota and phytopathogens (9, 33). *Sr*-T6SS is encoded in a 72-kbp-long cluster, of which the genetic organization resembles that of the phytopathogens *Ralstonia solanacearum* GMI1000 or the well-described *Acinetobacter baylyi* and *Acinetobacter baumannii* T6SS (34, 35). We recently showed that the *Sr*-T6SS drives bacterial community assembly during transmission from seed to seedling (9). *Sr*-T6SS encodes at least nine effectors and targets a broad diversity of seed-borne bacteria from six distinct bacterial families. Interestingly, these target strains display different susceptibility/resistance profiles to T6SS attacks, which correlates higher susceptibility with a closer phylogenetic distance and higher metabolic overlap to *S. rhizophila* (9). This observation suggested that the T6SS adapts to its microenvironment by targeting direct competitors first. However, the molecular explanation for such competitive efficacy and T6SS broad targeting of seed-borne bacteria like *Sr* is still unknown. Here, we investigated whether effectors could display activities against specific targets. We formulated three hypotheses to explain T6SS resistance: the target strain (i) does possess the cognate immunity of the deadly effector, (ii) does use an immunity-independent pathway, (iii) does not possess the molecular target of the effector, or the effector does not achieve its target. We also described the firing mode and regulation of the *Sr*-T6SS. Finally, the ecological role of the *Sr*-T6E-I repertoire was reevaluated through its origin and distribution in the bacterial diversity.

## RESULTS

### *S. rhizophila* encodes 13 putative effectors of the T6SS

**Fig 1:**
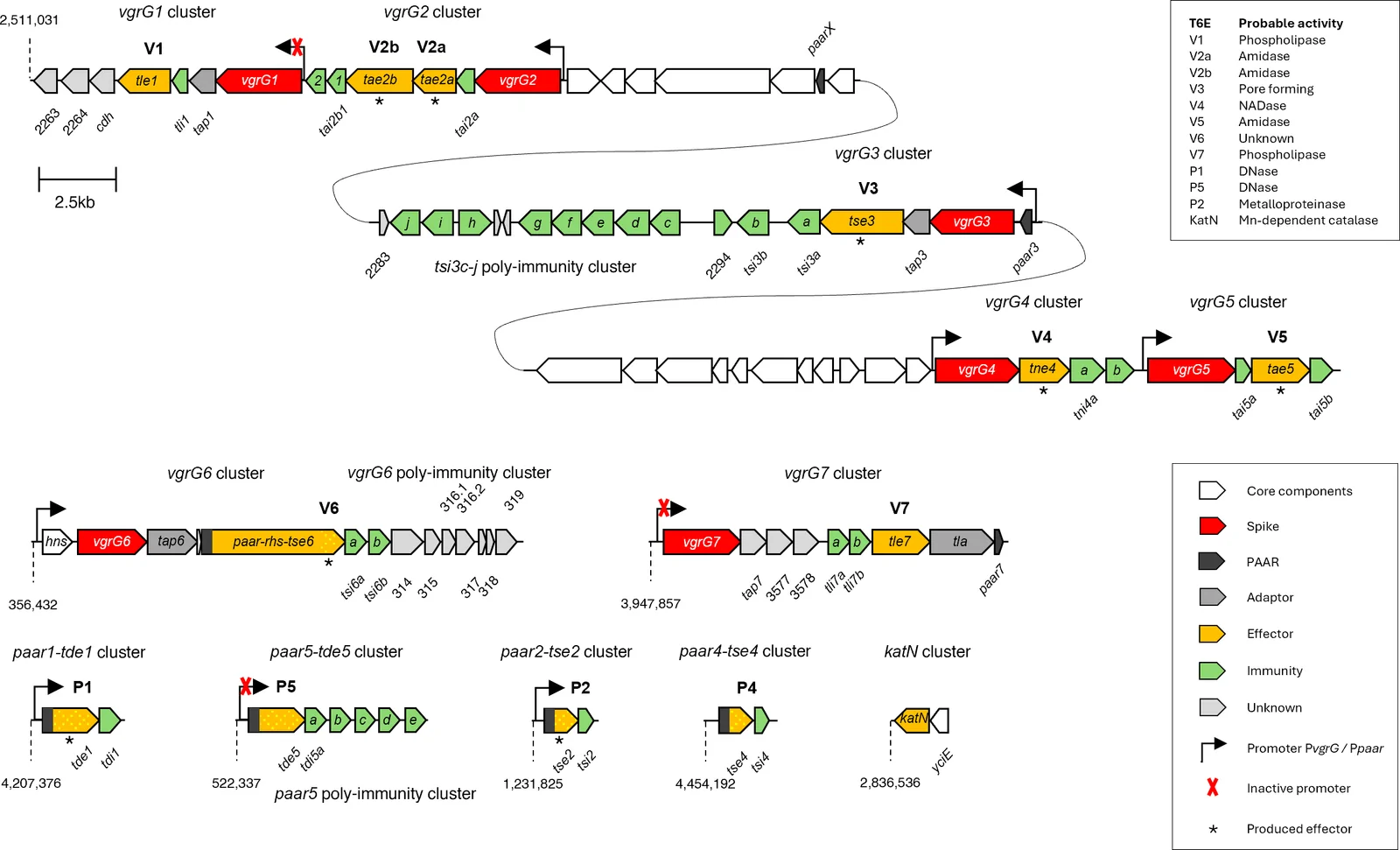
Genetic organization of *vgrG* and *paar-tse* gene clusters of *S. rhizophila* CFBP13503 T6SS. *Sr*-T6SS is encoded in a 72 kb gene cluster that is composed of 16 structural genes (white blocks) and 5 *vgrG* operons (*vgrG1-5*, red blocks), including adaptor genes (gray blocks), PAAR tip genes (*paarX*, *paar3,* and *paar7*, black blocks), 6 effector genes (orange blocks), and cognate immunity genes (green blocks). Six more orphan genetic clusters are found around the genome to be *vgrG* operons or *paar*-fused effector genes (*vgrG6*, *vgrG7*, *paar1-tde1*, *paar2-tse2*, *paar4-tse4*, *paar5-tde5* clusters). Another potential T6SS-associated effector is a manganese-dependent catalase KatN. Two poly-immunity clusters are associated with *vgrG3* and *vgrG6* clusters. Each cluster is likely governed by its own promoter upstream of *vgrG* or *paar* genes (black arrow). Genomic position for each cluster is indicated (vertical dashed line). See more details on cluster composition in Table S3.

We improve putative effector identification using *in silico* functional and structural analysis of proteins by structural predictions and protein-protein interaction modeling (36–38) and find four new additional T6Es than previously reported (33) (Table S1). T6Es are encoded in *vgrG1-7* operons or fused to orphan *paar1-2-4-5* genes (*paar-tse*). *vgrG1-5* operons are located within the 72 kb-long T6SS cluster, while *vgrG6-7* and *paar1-2-4-5-tse* are orphan and scattered around the genome (Fig. 1). These operons are predicted to be controlled by a dedicated promoter and typically contain one copy of *vgrG,* one *paar* tip, one adaptor (*tap*), one effector (*tse*), and its cognate immunity protein (*tsi*). Orphan *paar-tse* operons only contain the *paar*-fused effector (*paar-tse*) and the cognate immunity. Operons can also carry additional immunities.

***vgrG1*** operon encodes *vgrG1, tap1, tle1,* a putative phospholipase, and its cognate DUF3304-containing immunity *tli1*. Three other genes are encoded downstream on the same strand: *cdh* (CDP-diacylglycerol pyrophosphatase), *CLMFLP_11360,* a probable SapC homolog, and *CLMFLP_11355* of unknown function.

***vgrG2*** operon harbors *vgrG2* and two effector-immunity pairs of putative amidases: *tse2a-tsi2a* and *tse2b-tsi2b1,* plus an additional homologous immunity *tsi2b2. tse2a* is predicted to encode an Slt-like transglycosylase, and *tse2b* is predicted to encode a Zn-dependent DD-peptidase.

***vgrG3*** operon encodes *vgrG3*, *tap3,* and *tse3,* followed by its cognate immunity *tsi3a*. Tse3 is predicted to possess an N-terminal MIX-III domain and a BTH_I2691 domain specific to pore-forming activity (39). Strikingly, *tsi3* is followed by 12 homologous genes (*tsi3b-j*) in a 14 kb region. *CLMFLP_11510,* which is found in this cluster, encodes a putative FGE-fold protein known to activate sulfatases in eukaryotes and already found as a widespread T6E immunity (40).

***vgrG4*** operon possesses *vgrG4* and *tse4,* which encodes a FIX-Ntox15 domain-containing protein linked to the PoNe toxin family. Our analysis indicated that the Tse4 C-terminal domain is a probable NADase toxin similar to Tse6 (PDB 4ZV0) and Tne2 (PDB 6B12) in *Pseudomonas* strains (41). Tse4 is predicted to be blocked by a cognate DUF1911 domain-containing immunity Tsi4a but not Tsi4b encoded in tandem.

***vgrG5*** operon is organized with *vgrG5*, *tai5a*, *tae5,* and *tai5b*. Specifically, Tae5 is composed of multiple domains, notably Tae5-Nt and Tae5-Ct. Tae5-Nt blasts with the endolysin Enc34 (PDB 7Q47) and the transglycosylase MltD (PDB 4C5F). Tae5-Ct blasts with the N-terminal amidase domain of a bifunctional glutathionylspermidine (Gsp) synthase/amidase GspSA (PDB 3A2Y) found in *Escherichia coli* (42). The two signal peptide-containing Tsi5 immunities seem to each interact with Tae5 at specific domains, underlining a potential bifunctionality for Tse5 (43).

***vgrG6*** operon is composed of *hns* upstream *vgrG6*, *tap6,* and the fused *paar-rhs-tse6* gene, followed by two cognate immunities *tsi6a and tsi6b*. It also carries seven other genes (*CLMFLP_01565-01595*) with unknown functions. Surprisingly, the structural prediction of VgrG6 trimer shows a longer tip compared to the other identified VgrG trimers, suggesting a specific use (Fig. S1). Tse6 is the C-terminal domain of the fused protein PAAR-Rhs (44, 45) and does not possess any known functional domain. Interestingly, the genomic region from *tse6* (Rhs-Ct, *CLMFLP_01555*) to *CLMFLP_01590* displays a lower GC% than the *S. rhizophila* T6SS cluster average (56 GC% against 64 GC%). Furthermore, the corresponding genes encode putative Knr4/Smi1, Imm26, or LRR domain-containing proteins that are associated with antitoxin and immunities (46–48).

***vgrG7*** operon carries *vgrG7*, *tap7* (DUF4123), *CLMFLP_17965,* and *CLMFLP_17970* of unknown function, *tli7a*, *tli7b*, *tle7*, *tla,* and *paar7*. Only Tli7a is predicted to inhibit the phospholipase activity of Tle7 with the highest confidence, although with a score under the acceptable threshold (Table S4). Unlike Tli1, Tli7a and Tli7b do not possess a signal peptide sequence, suggesting that they have a different role than phospholipase inhibition in the periplasm (49).

***paar1-tde1*** and ***paar5-tde5*** operons encode the homologous DNase effector-immunity pairs. Orphans *paar1-tde1* and *paar5-tde5* genes consist of the fusion of PAAR to an Ntox15-containing C-terminal domain (50). The cognate immunity (*tdi1* or *tdi5*) is located directly downstream, but *paar5-tde5* possesses four additional copies of which the first two are predicted to also interact with Tde5. Conversely, neither Tdi1 nor Tdi5a, b, c, and d are predicted to interact with Tde5 or Tde1, respectively.

Orphan ***paar2-tse2*** operon contains the fused PAAR2-Tse2 putative effector and its cognate immunity Tsi2. Structural comparison of Tse2 suggests that it functions as an MMP-like metalloproteinase, such as the Karilysin (PDB 2XS3), secreted by the T9SS of *Tannerella forsythia* (51). The cognate immunity carries a signal peptide and blasts with the PotempinA protein, also found in *Tannerella* as a pro-peptide fused with the toxic domain and cleaved upon secretion (PDB 8B2Q) (52). It has been shown that such an effector activity, also found in *Acinetobacter baylyi* and *Pseudomonas aeruginosa*, di*s*rupts cell morphology in the target bacterium (34, 53). A homolog of PAAR2-Tse2 is found elsewhere in the genome, in the ***paar4-tse4*** cluster that possesses the same structure as the *paar2* cluster, with the difference that its GC% is much lower (55% vs 64%).

A manganese-dependent catalase-encoding gene, ***katN****,* potentially secreted by the T6SS, was also identified. KatN is known to increase survival under oxidative stress in EHEC and *Burkholderia* (5, 54).

Each effector is named hereafter with a single letter, followed by a number corresponding to the operon they are attached to, as follows: V1 (for *tle1* from *vgrG1* operon), V2a and V2b (from *vgrG2* operon, effector Tse2a or Tse2b), V3, V4, V5, V6, V7, P1, P2, P4, and P5 (Table S1).

### *Sr*-T6SS potentially secretes 9 out of 13 effectors

To know whether *S. rhizophila* WT uses its whole T6E repertoire, we tested the genetic expression of each *vgrG* operon through fluorescent transcriptional reporters of predicted promoters (P*vgrG* or P*paar*) that we validated by their direct detection using high-resolution tandem mass spectrometry-based proteomics (Table 1; Table S8). Transcriptional reporters showed that all but P*vgrG1*, P*vgrG7,* and P*paar5* are active in both exponential and stationary growth phases, but are stronger in the stationary phase during TSB10 liquid culture (Fig. S2A). P*vgrG2*, P*vgrG5*, P*hns* (*vgrG6* operon), and P*paar1* showed the strongest activity, which is correlated with associated protein abundances measured by mass spectrometry in stationary phase cells (Table 1). The expression of both phospholipases-encoding clusters *vgrG1* and *vgrG7,* as well as *paar5-tde5* clusters, was absent in the tested conditions, as no mRNA was detected in RT-PCR of stationary growth phase cells (Fig. S2B). Since high NaCl concentration has been found to up-regulate T6SS genes in the closely related strain DSM14405 (55), we tested its effect on *vgrG*/*paar* promoters, but no difference or a lower activity was observed when exposed to NaCl 1% compared to mock TSB10 (NaCl 0.05%) in the exponential phase (Fig. S2C). Despite no or very poor detection of secreted effectors in the supernatant (Fig. S2D), we were able to detect the production of 9 out of 13 T6Es, as well as 6 dedicated VgrGs in the whole cell proteome of stationary growth phase cells (Table 1). Additionally, P5 and VgrG1 were detected at a very low level, suggesting a potential secretion under inducible conditions. Four additional immunities, Tai2b2, Tsi6b, 314, Tdi5b, and Tdi5d, but no other immunities associated with *vgrG3*, *vgrG4*, *vgrG7,* and *vgrG6* clusters were detected in the overall proteome, also suggesting an independent and conditional regulation.

**TABLE 1:** T6SS effector-associated proteins detected in the stationary growth phase *S. rhizophila* CFBP13503 total proteome^*a*^

Category	Accession number	Protein	Functional description	Predicted function	MW (kDa)	µSC	sd-SC	µNSAF	sd-NSAF
VgrG spike	WP_141057885.1	VgrG1	Type VI secretion system Vgr family protein	T6SS spike	97.5	3.5	1.3	4	1.3
	WP_141058098.1	VgrG2	Type VI secretion system Vgr family protein	T6SS spike	97	27.8	4.3	29	4.4
	WP_141059348.1	VgrG3	Type VI secretion system Vgr family protein	T6SS spike	97.2	18.8	6.1	19	6.2
	WP_392731641.1	VgrG4	Type VI secretion system Vgr family protein	T6SS spike	98.4	30	7.8	30	7.9
	WP_141059353.1	VgrG5	Type VI secretion system Vgr family protein	T6SS spike	101.9	34.3	8.2	34	8.1
	WP_141058144.1	VgrG6	Type VI secretion system Vgr family protein	T6SS spike	81.7	31	5.7	38	7
T6E	WP_141057882.1	V2a	Hypothetical protein	M23 family Slt-like transglycosylase	50	7.5	1.7	15	3.5
	WP_141057883.1	V2b	SH3 domain-containing protein	Zn-dependent DD-peptidase	79.8	22.5	4.4	**28**	5.6
	WP_141059346.1	V3	BTH_I2691 family protein	Pore-forming toxin	99.3	17.3	12.6	17	12.7
	WP_219994946.1	V4	Hypothetical protein	PoNe family NADase	60.7	2.3	1.5	4	2.5
	WP_141059354.1	V5	TIGR02594 family protein	Bifunctional glutathionyl- spermidine synthase/amidase	68.3	8.3	1.3	12	1.8
	WP_141058143.1	V6	RHS repeat-associated core domain-containing protein	Unknown	172.2	15.8	8.3	9	4.8
	WP_141057660.1	P1	Polymorphic toxin type 15 domain-containing protein	DNase	66.8	6.8	4.6	10	6.9
	WP_141057258.1	P2	PAAR domain-containing protein	Metalloproteinase	39.4	7	1.4	18	3.6
	WP_141058559.1	P5	Polymorphic toxin type 15 domain-containing protein	DNase	66.4	2.3	2.6	3	4
T6I	WP_161578710.1	Tli1	DUF3304 domain-containing protein	V1 immunity	18.8	1.5	0.6	8	3.1
	WP_141057881.1	Tai2a	Ivy c-type lysozyme inhibitor	V2a immunity	20.8	9	2.2	43	10.4
	WP_141057884.1	Tai2b1	Decarboxylase	V2b immunity	20.9	10.8	2.1	51	9.9
	WP_181406176.1	Tai2b2	Decarboxylase	V2b homologous immunity	22.4	0.3	0.5	1	2.2
	WP_141059345.1	Tsi3a	DUF6708 domain-containing protein	V3 immunity	38.7	29.8	4.8	77	12.4
	WP_141059351.1	Tni4a	DUF1911	V4 immunity	42.6	9.3	4	22	9.5
	WP_137189658.1	Tai5a	Hypothetical protein	V5-Nt immunity	18.5	1.5	0.6	8	3.1
	WP_137189660.1	Tai5b	M949_RS01915 family surface polysaccharide biosyn. protein	V55-Ct immunity	25.9	2	1.8	8	7
	WP_141058142.1	Tsi6a	Hypothetical protein	V6 immunity	25.5	9	1.4	35	5.5
	WP_141058141.1	Tsi6b	Hypothetical protein	V6 homologous immunity	26.2	1	1.2	4	4.4
	WP_141058140.1	314	LRR domain-containing protein	Nuclease immunity	38.4	2.3	0.5	6	1.3
	WP_141057661.1	P1tdi1	GAD- like domain-containing protein—DUF1851	P1 immunity	25.8	6.5	2.6	25	10.2
	WP_141057257.1	P2tsi2	Hypothetical protein	P2 immunity	18.6	10.8	2.6	58	14.1
	WP_141058558.1	P5tdi5a	GAD- like domain-containing protein—DUF1851	P5 immunity	25.9	4.8	3.9	18	14.9
	WP_141058557.1	P5tdi5b	GAD- like domain-containing protein—DUF1851	P5 homologous immunity	25.8	2.8	2.8	11	10.7
	WP_392732912.1	P5tdi5d	GAD- like domain-containing protein—DUF1851	P5 homologous immunity	25.6	3.5	3.7	14	14.5
Adaptor	WP_038688001.1	PAAR3	Hypothetical protein	VgrG3-associated PAAR tip	12.8	46.8	7.1	364	55.6
	WP_181406191.1	Tap6	Hypothetical protein	V6 adaptor	59.5	12.8	0.5	21	0.8
	WP_258317547.1	Tap3	DUF4123 domain-containing protein	V3 adaptor	32.2	11.8	4.3	36	13.5

^*a*^ MW, molecular weight; SC, spectral count; NSAF, normalized spectral abundance factor (expressed as SC/MW × 10^2^); µ, mean; sd, standard deviation of four biological replicates.

### *Sr*-T6SS is constitutive and highly dynamic

**Fig 2:**
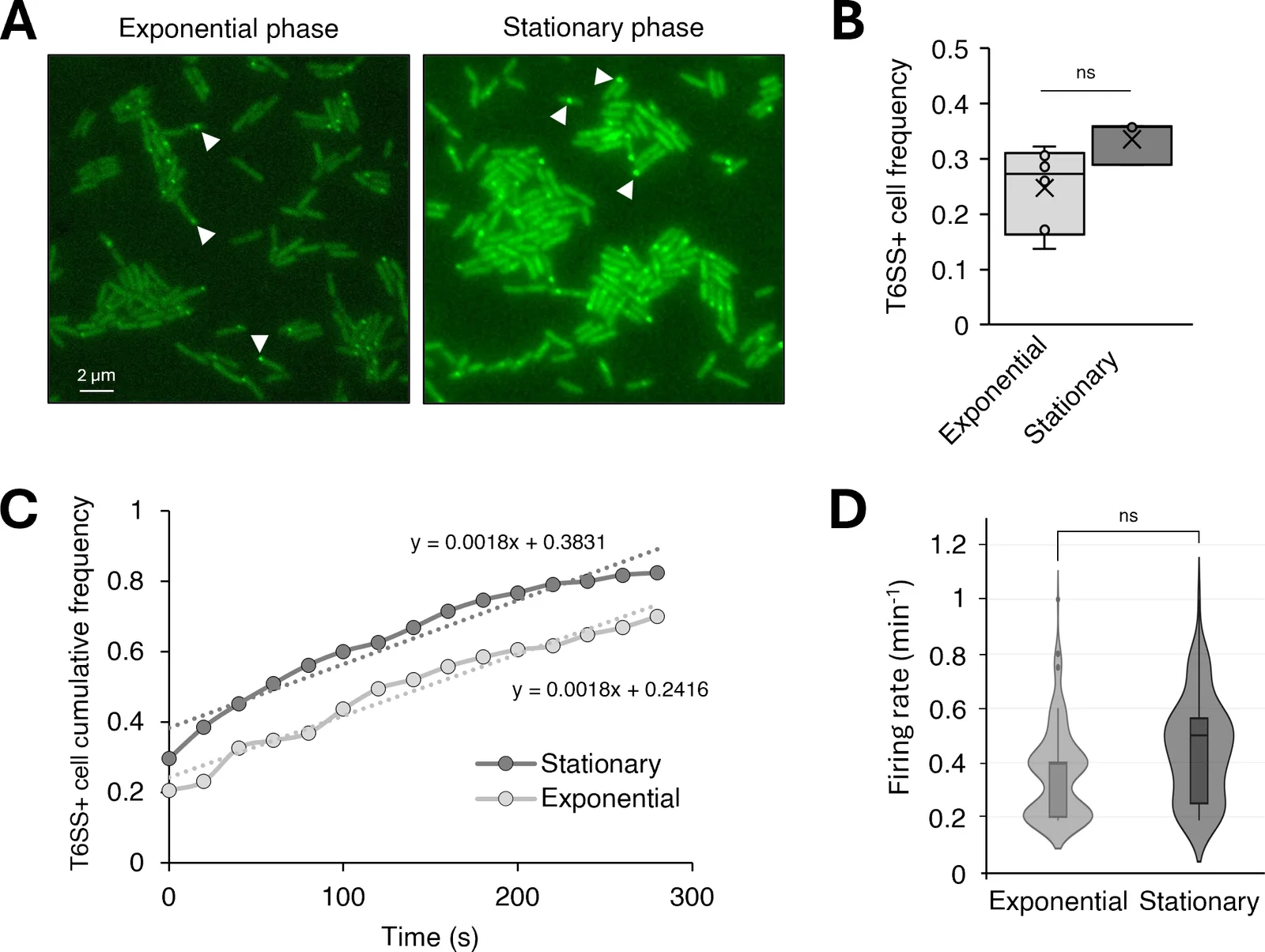
T6SS dynamics of *S. rhizophila* CFBP13503. (**A**) Microscopy illustration of TssB::sfGFP-expressing *S. rhizophila* B-GFP strain grown in TSB10 for 5 h (exponential) or 17 h (stationary). Fluorescence signal was adjusted to the same threshold. (**B**) T6SS+ cell frequency in exponential and stationary B-GFP populations. The cell number with a T6SS focus (white arrow) is counted and divided by the total cell number to calculate T6SS+ cell frequency. Boxplot representation combines six and three technical replicates, cumulating 829 and 637 for exponential and stationary phase cells, respectively. (**C**) Cumulated frequency of T6SS+ cells in exponential or stationary growth phases during 4.5 min time-lapse. The time-lapse consisted of 15 frames taken every 20 s with a GFP filter and resulted in the T6SS detection over a total of 190 exponential and 623 stationary phase cells. The value for each time frame is the T6SS+ cell frequency cumulated with the previous time frame. (**D**) T6SS firing rate in exponential or stationary phase cells. The number of T6SS assembly events was measured using a time-lapse of 20 s intervals for 5 min. The violin plot was drawn with data from seven and three independent biological replicates, gathering 255 exponential and 109 stationary phase cells, respectively. Means were compared with a Wilcoxon test (ns, *P* > 0.05).

To better understand how the T6SS is functioning against target cells, we first described its dynamics with a translational reporter strain B-GFP, where the major sheath subunit TssB was fused to the sfGFP, allowing for T6SS monitoring by fluorescence microscopy (Fig. S3). The TssB::sfGFP fusion produced a uniform and diffuse fluorescence in the cell, indicative of a constitutive and homogeneous T6SS expression, as well as foci corresponding to assembled or contracted sheaths (Fig. 2A). We observed that the *Sr*-T6SS never assembles throughout the whole cell but instead mostly forms a single, polar, short, and functional focus (Fig. S3). We measured that approximately 20% to 30% of cells simultaneously activate the T6SS in a *S. rhizophila* population (Fig. 2B). However, a 5 min time-lapse experiment showed that 70% and 80% of the population grown in exponential and stationary phases, respectively, can assemble a T6SS with a high firing dynamic (Fig. 2C and D). A longer time-lapse experiment confirmed that the producing cell frequency remains stable over time while the individual producing cell frequency is dynamic (Fig. S3).

### Cell death phenotype from *Sr*-T6SS activity is specific to the target strain

**Fig 3:**
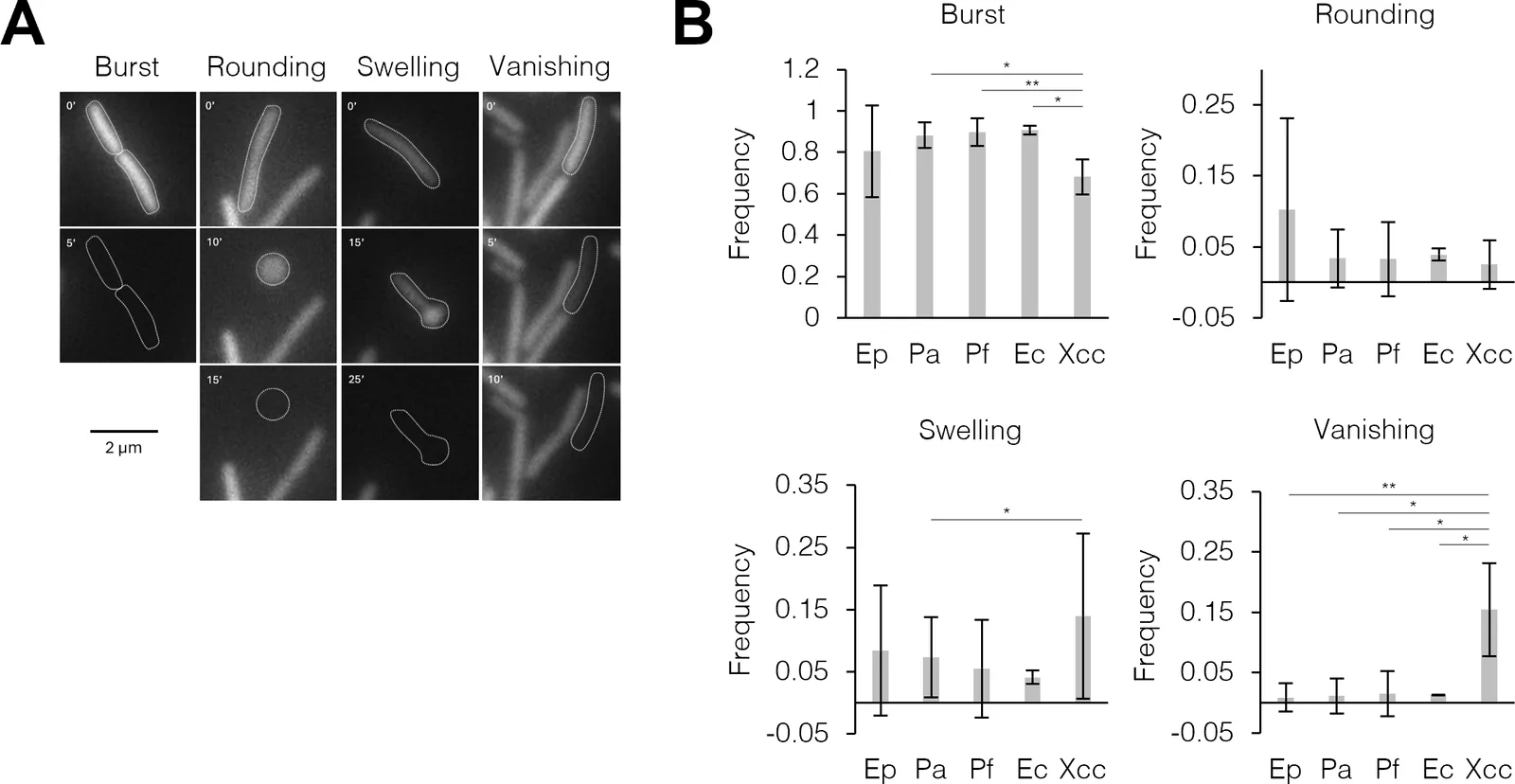
*S. rhizophila* CFBP13503 T6SS-induced cell death phenotypes. (**A**) Cell death phenotypes induced by T6SS activity on *E. coli* mCherry target cells when competing with *S. rhizophila* WT. Time-lapse images were taken every 5 min. The time for the observation of the death appearance is indicated in minutes. (**B**) Frequency of cell death phenotypes in different target strains when competing with *S. rhizophila* for 1 h. *Ep, Erwinia persicina* CFBP8803; *Pa, Pantoea agglomerans* CFBP13505; *Pf, Pseudomonas fluorescens* CFBP13510; *Xcc, Xanthomonas campestris* pv. *campestris* 8004; *Ec, Escherichia coli* W3110. Values combine three to five biological replicates. One specific cell death phenotype frequency is compared between each strain with a Wilcoxon test (*, *P* < 0.05; **, *P* < 0.005).

Since the cell death phenotype is dependent on the effector activity (34), we figured out how the T6Es of *S. rhizophila* kill target strains. Using time-lapse microscopy of a competition assay between *S. rhizophila* and fluorescently labeled target strains, we observed four major cell death phenotypes produced by the T6SS activity, among the cell burst (instant or brutal disappearance of the target cell), rounding, swelling, and vanishing (gradual fading of the shine until the disappearance of the target cell) (Fig. 3A; Supplemental file). This observation suggests that multiple effectors are secreted and responsible for the *Sr*-T6SS antibacterial effect. Strikingly, we observed that the average cell death frequency varies among target strains, with *Xcc*, *Erwinia perscicina*, *Pantoea agglomerans*, *Pseudomonas fluorescens,* and *E. coli* presenting a higher percentage of vanishing, rounding, or bursting, respectively (Fig. 3B). This observation suggests that the *Sr*-T6SS is acting differently on different target strains.

### T6SS effector repertoire provides target strain specificities

We observed that seed-associated bacteria display a differential susceptibility profile to *Sr*-T6SS attack (Fig. S4), whereas *S. rhizophila* itself is largely resistant to attacks from others (9). To explain *Sr*-T6SS susceptibility in the seed microbiota, we tested three hypotheses.

**Fig 4:**
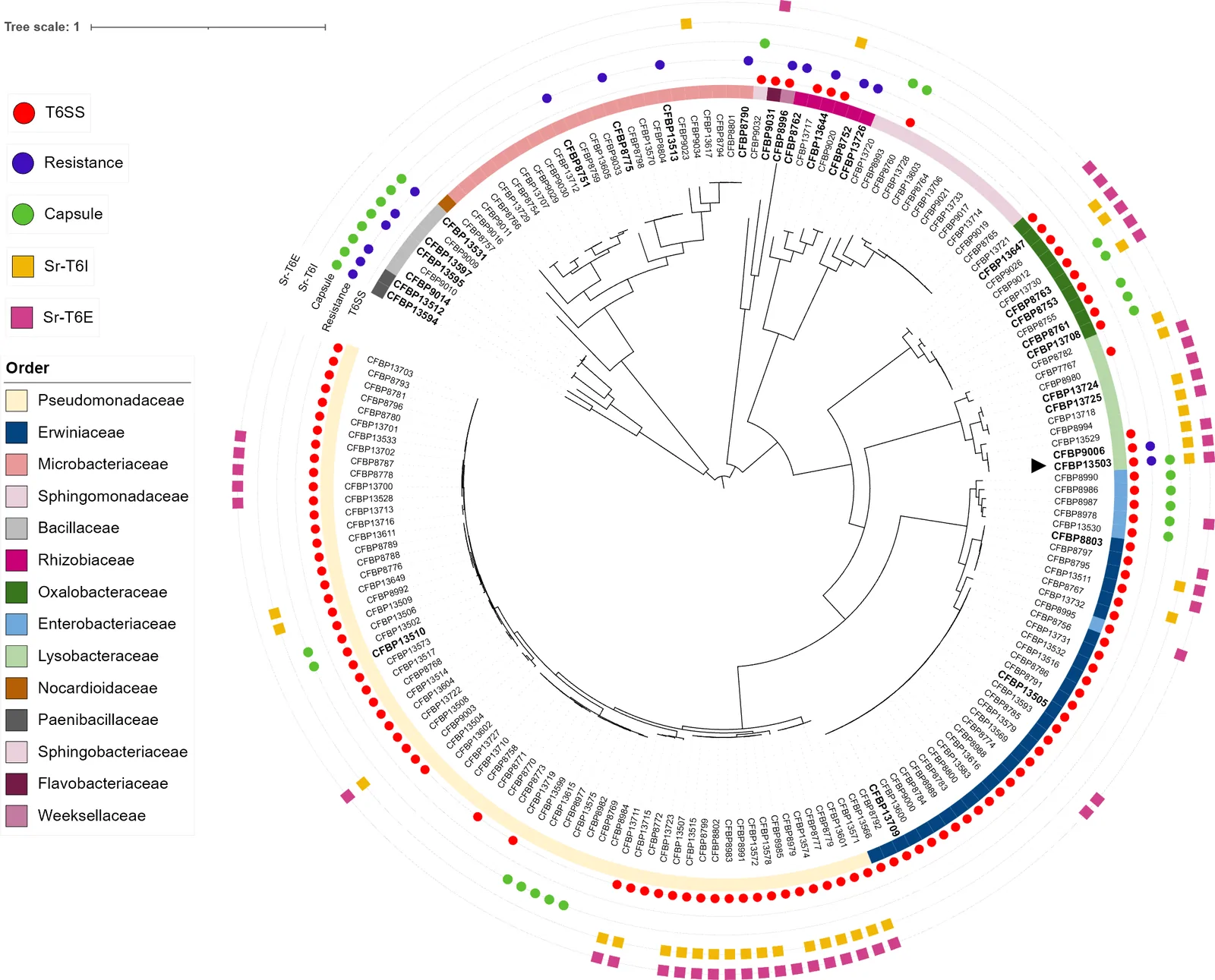
*S. rhizophila* CFBP13503 T6SS effector (Sr-T6E) and immunity (Sr-T6I) gene distribution, T6SS resistance, and capsular gene distribution in 178 seed-associated bacteria. Maximum likelihood phylogenetic tree was constructed by GToTree (option IQ-TREE 1,000 bootstrap) using 74 marker genes from a data set of 178 genomes of seed-associated bacterial strains of rapeseed, radish, and bean. Bacterial strains are denoted by their CFBP number and are color-coded according to their phylogenetic family. *S. rhizophila* CFBP13503 is indicated by an arrow. T6SS presence determined by TssB or TssC detection is indicated by a red circle (first, internal layer). *Sr*-T6SS susceptibility profile of selected strains (bold) is indicated by a blue circle when resistant, determined by a mortality <1 log_10_ during a competition assay (second layer). The detection of capsular genes by CapsuleFinder is indicated by a green circle (third layer). The presence of at least one *Sr*-T6I ortholog is indicated by an orange square (fourth layer), and the presence of at least one *Sr*-T6E ortholog is indicated by a purple square (fifth, outer layer). Absence of a sign indicates the absence of indicated features.

**Fig 5:**
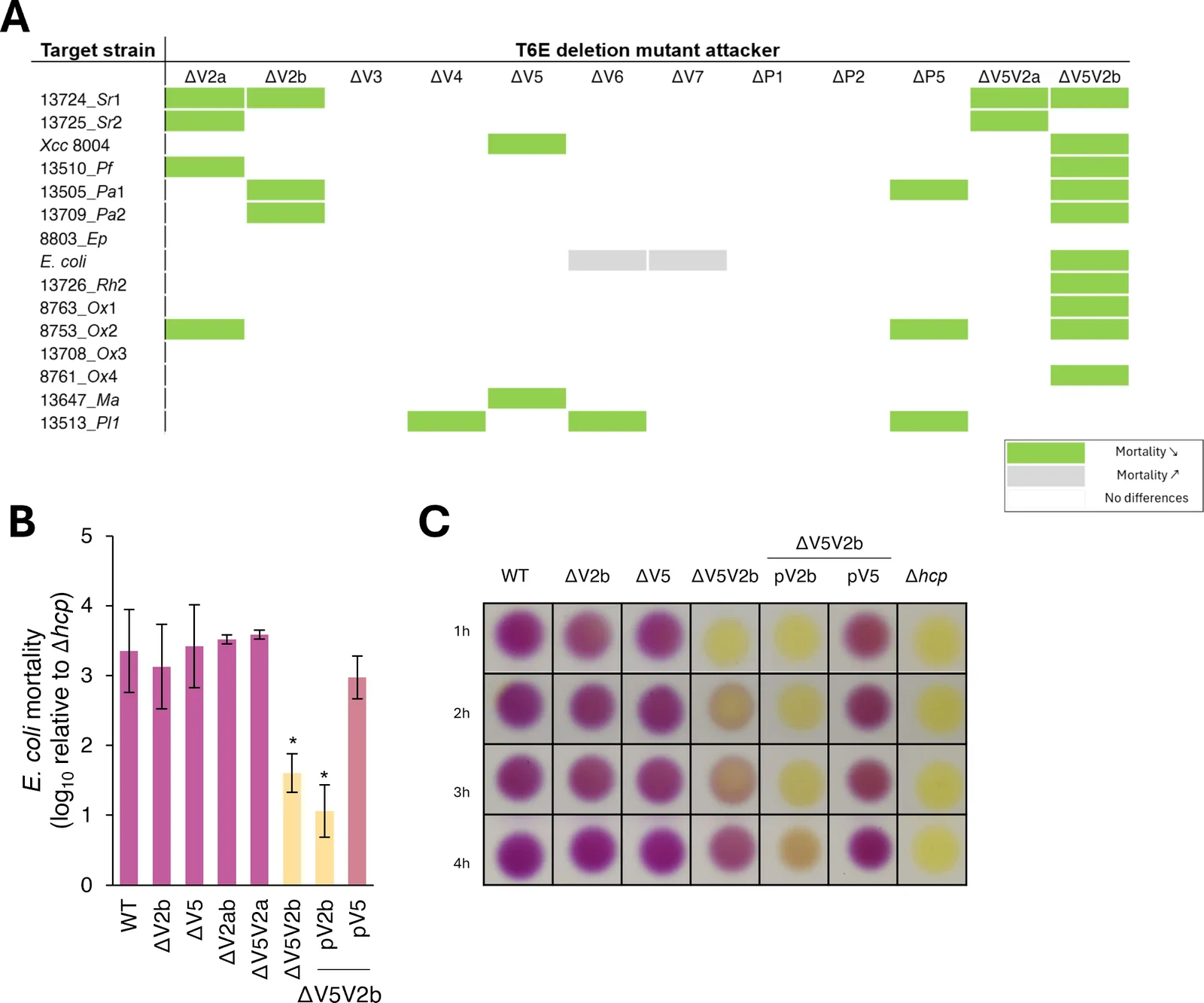
Strain-specific targeting of *S. rhizophila* CFBP13503 T6SS effectors (T6E) and role of two putative amidases in *Sr*-T6SS antibacterial activity. (**A**) Heatmap of the relative impact of *S. rhizophila* T6E deletion mutants compared to the WT strain (attackers) on mortality of different target strains during a quantitative competition assay using the survivor growth kinetic (SGK) method or the colony-forming unit (CFU) counting. Target strains are indicated by the CFBP number as coded in Table S1. The target strain mortality is calculated as the log_10_ change in cell recovery compared to the T6SS deletion mutant Δ*hcp*. Data combine three or four biological replicates detailed in Fig. S6 and S10. Differences between WT and T6E deletion mutants were assessed using a Wilcoxon test: green, gray, or white boxes indicate a significant mortality decrease, increase (*P* < 0.05), or no difference (*P* > 0.05), respectively. (**B**) Quantitative competition assay between *E. coli* W3110 target and *S. rhizophila* WT, single, double putative amidase deletion mutants, or ΔV5V2b complemented strains (pV2b and pV5) as attacker strains. *E. coli* mortality is calculated as the log_10_ change in cell recovery compared to the T6SS deletion mutant Δ*hcp* using the SGK method. Values are derived from four biological replicates, where bars represent the mean and error bars are the standard deviation. Statistical comparisons were performed using a Wilcoxon test (*P* < 0.05: *). (**C**) Qualitative competition assay between *E. coli* W3110 target and *S. rhizophila* WT, single, double putative amidase deletion mutants, or complemented strains as attacker. Target cell lysis was revealed by the addition of chlorophenol red-β-D-galactopyranoside (CPRG) after 1, 2, 3, and 4 h based on the lysis-associated β-galactosidase assay (LAGA) method. A yellow color indicates no target cell lysis, and a purple color indicates a high level of lysis. Intermediate colors indicate a low to medium level of target cell lysis. *S. rhizophila* Δ*hcp* strain is used for a negative control of T6SS antibacterial activity.

- Since T6SS resistance can be explained by the presence of orthologous immunities alone (13, 14), we first searched for *S. rhizophila* immunity (*Sr*-T6I) homologues in 177 genomes of strains isolated from radish, rapeseed, or bean seeds (9). We detected at least one orthologous *Sr*-T6I in 19% of strains (*n* = 33) (Fig. 4; Fig. S5). We then used AlphaFold3 modeling to predict the inhibition of these orthologous T6Is against the cognate *Sr*-T6Es and measured 45% of target strains potentially having a positive interaction with at least one effector (*n* = 15), only representing 8% of total seed-associated bacteria (Table S5). Despite the presence of cognate immunities to *Sr*-T6Es, we measured that *Sr2* and *Ox1* target strains are not fully resistant to *Sr*-T6SS attacks. Only *S. rhizophila* CFBP9006, the closest strain to CFBP13503, possesses all *Sr*-T6Is but not Tsi6 and is fully resistant. This analysis showed that orthologous immunities do not drive *Sr*-T6SS susceptibility in plant seed microbiota.
- Capsule production has also been reported to be involved in T6SS resistance. We thus searched for capsular genes in the strain diversity (15, 56). Among the 177 genomes, we detected capsular genes in 16% of strains representing six families (Fig. 4). However, we observed no systematic correspondence between *Sr*-T6SS resistance and capsular gene presence in 30 selected strains, notably in Gram-negative bacteria. This analysis showed that capsular genes are weakly distributed and are not the major *Sr*-T6SS resistance pathway in seed-associated bacteria.
- To investigate whether T6SS susceptibility can be explained by a specific *Sr*-T6E activity in the target strain (i.e., to explain T6SS resistance by the absence or a reduced *Sr*-T6E activity), we produced every single-effector deletion mutant of *S. rhizophila* CFBP13503 and we performed competition assays against 13 susceptible strains isolated from seeds, representing the six distinct bacterial families *Pseudomonadaceae, Microbacteriaceae, Rhizobiaceae, Enterobacteriaceae, Lysobacteraceae,* and *Oxalobacteraceae* (9). This experiment revealed target strain specificities with low but significant target mortality reduction when competing with effector deletion mutants compared to the WT strain (Fig. 5A; Fig. S6). We notably observed the impact of the three putative amidases V2a, V2b, and V5 single deletion on targeting the members of the *Gammaproteobacteria,* such as *S. rhizophila*, *Xanthomonas campestris* pv. *campestris, P. fluorescens,* and *P. agglomerans*. We also observed that deletion mutants have no effect on the *Sr*-T6SS antibacterial activity against some strains (e.g., *Ep*, *Rh2*, *Ox1*, *Ox3*, *Ox4*, *Ec*), showing a general synergetic effect of effectors. More importantly, this observation showed that effector deletions do not impair the T6SS activity (Fig. S7).

Inversely to *Ox1*, *Ox3,* and *Ox4*, *Ox*2 mortality is reduced when competing with V2a and P5 deletion mutants (0.51 ± 0.09 log_10_ and 0.46 ± 0.20 log_10_, respectively). *Massilia* mortality is also reduced against the V5 deletion mutant (0.55 ± 0.08 log_10_). Since the cited strains belong to the same family, this result suggests that each effector potency is defined by the strict target strain specificity.

Surprisingly, P5 effector deletion impacted the killing of species from different families (*Ox*2, *Pl*1, and *Pa*1). Since we could not detect its expression nor its production in liquid culture, the negative impact of its deletion on the killing efficiency should imply that it is used by the T6SS. *Ox*2 and *Pa*1 carry one T6SS cluster and *Pa1* displayed lysis-associated antibacterial activity against *E. coli in vitro* (Fig. S8A), raising the hypothesis that P5 could be induced upon an attack from aggressive competitors (57, 58). Finally, we measured P5 promoter (P*paar5-tde5*) induction in the stationary growth phase on solid medium. A higher P5 promoter induction was observed when competing with other strains, independently of its aggressive behavior, compared to the absence of the target strain (Fig. S8B).

The survival of the Gram-positive bacterium *Plantibacter* (*Pl*1) was affected by three effectors: P5 (0.43 ± 0.15 log_10_ mortality reduction), V4 (0.45 ± 0.17 log_10_ mortality reduction), and V6 (0.57 ± 0.26 log_10_ mortality reduction). We hypothesized that V6 could target Gram-positive bacteria or organisms with thick cell envelopes, based on the very long predicted structure of the VgrG6 spike. In our strain selection, no other target mortality was reduced by V6 deletion, supporting the hypothesis that it is indeed specifically employed against Gram-positive bacteria. These results suggest that *Sr*-T6SS susceptibility profiles can be explained by specific effectors.

### T6SS killing mostly depends on two amidases

To test if the deletion of multiple effectors could have more impact on the *Sr*-T6SS killing activity, we produced the double mutants of the most expressed amidases V2a, V2b, and V5. Strikingly, the double mutant V5V2b was highly impaired in killing *E. coli* compared to WT or single-deletion mutants, while its activity is restored when complemented with V5 (Fig. 5B). Further investigation suggested that the double mutation affects the ability to produce early bursting of the target cells (Fig. 5C), whereas not a single-effector deletion mutant displays this phenotype (Fig. S7). Microscopy observation of a competition assay confirmed this result (Fig. S9). Interestingly, this double mutant was also impaired in killing almost all selected seed-associated strains (Fig. 5A; Fig. S10). Taken together, these results indicate that not a single effector but the two amidases V2b and V5 play a major role in the antibacterial activity of the *S. rhizophila* T6SS.

### Distribution and conservation of T6E-I repertoire

**Fig 6:**
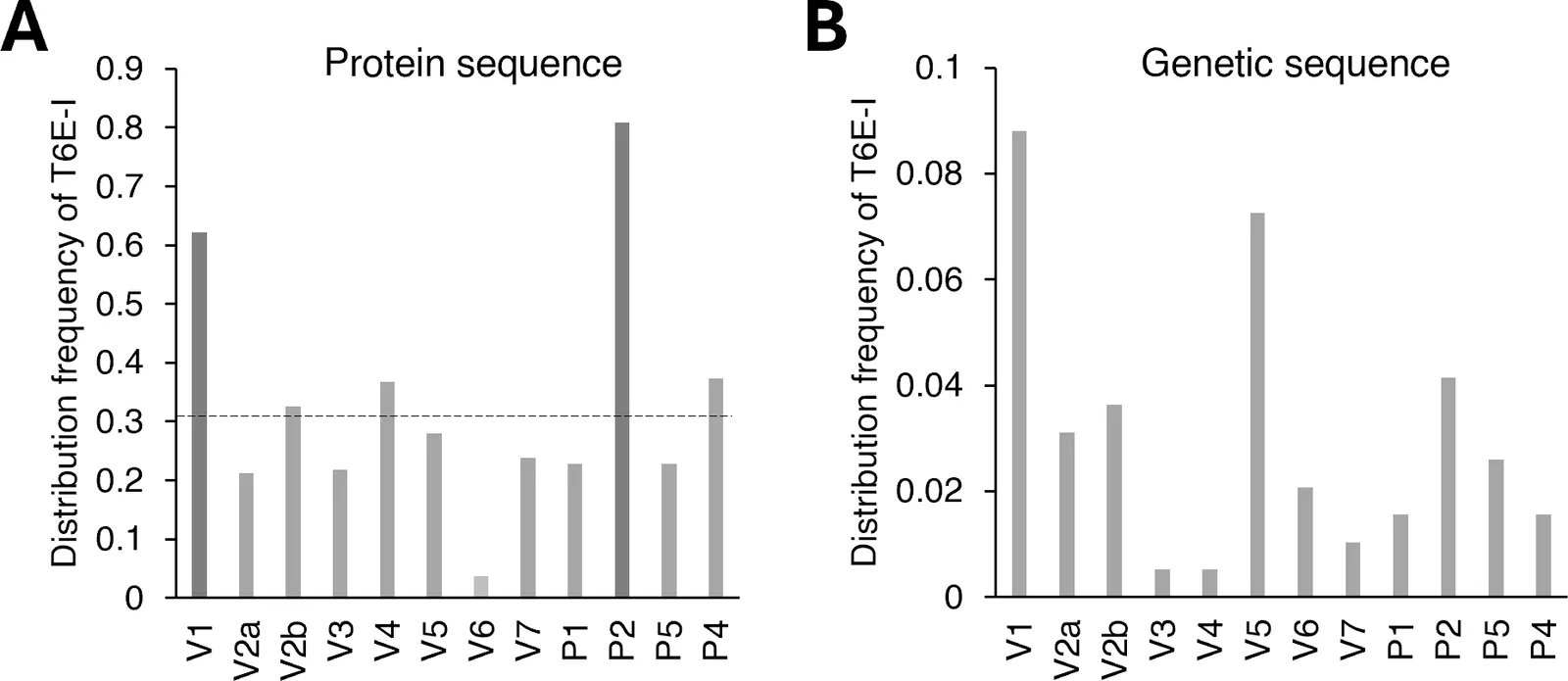
Distribution of *S. rhizophila CFBP13503* T6SS effector and immunity pairs (Sr-T6E-I) in *Stenotrophomonas* genus diversity. (**A**) Distribution of *Sr*-T6E-I pairs in *Stenotrophomonas* diversity based on amino-acid sequence from CFBP13503 strain. Each Sr-T6E-I pair was subjected to a protein blast using Cblaster (CAGECAT, August 2025). TssB query was used to count the number of T6SS+ strains (*n* = 193), which was the reference to normalize T6E distribution frequency among *Stenotrophomonas* strains. Average Sr-T6E-I distribution frequency is indicated with a dashed line. (**B**) Distribution of *Sr*-T6E-I pairs in *Stenotrophomonas* diversity based on genetic sequence from CFBP13503 strain. Each Sr-T6E-I pair was subjected to a nucleotide blast using Blastn (NCBI, August 2025). T6SS+ strain number previously found with TssB blast was used to normalize the distribution frequency.

To gain more insights into the ecological role of the *Sr*-T6E-I repertoire, we searched for its origin and distribution in the bacterial diversity. We first tested the presence of each *Sr*-T6E-I pair in the *Stenotrophomonas* diversity based on the amino-acid sequence and found that they are equally distributed, ranging from 20% to 40% of *Stenotrophomonas* strains (out of 193 strains) with an overrepresentation of V1 (60%) and P2 (80%) (Fig. 6A). This distribution is dramatically reduced (<10%) when the genetic sequence of the T6E-I pair is used as a query (Fig. 6B).

We then searched *Sr*-T6Es homologs in seed-associated bacteria and found that only 26% of strains possess at least one, with the phospholipases V1–V7, the pore-forming V3, and the DNases P1–P5 as the most shared (Fig. S5; Table S6). By contrast, using the NCBI database we found that *Sr*-T6Es are associated with 117 different genera, mostly representing Gammaproteobacteria and Betaproteobacteria (Table S7). V2b and V5 are distributed in 16 and 30 genera, respectively. (Table S7). However, only *Burkholderia ubonensis* and *S. rhizophila* strains were found to carry both effectors.

## DISCUSSION

**Fig 7:**
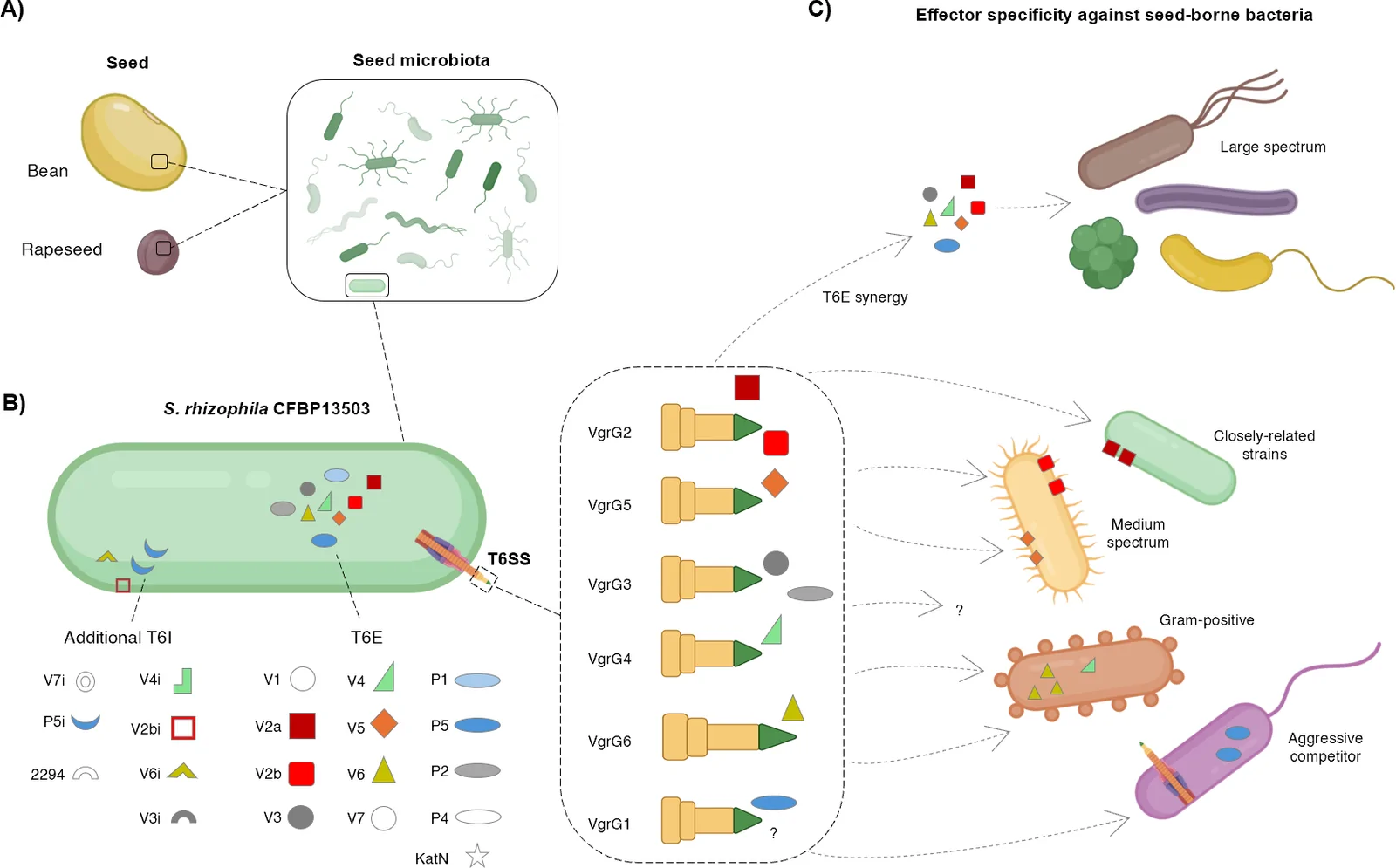
Model of T6SS-mediated killing by *Stenotrophomonas rhizophila* CFBP13503 T6SS effector repertoire in the seed microbiota. (**A**) Seeds carry diverse bacteria—referred to as the seed microbiota—that engage in interbacterial competition for space and nutrients or for plant protection. (**B**) *S. rhizophila* CFBP13503 is a seed-associated bacterium that possesses a T6SS-dependent antibacterial activity against a wide range of species. *Sr*-T6SS genetic basis encodes 13 putative effectors (T6E), of which 9 are produced (V2a, V2b, V3, V4, V5, V6, P1, P2, P5) and potentially secreted through 6 different VgrG1-6. (**C**) *S. rhizophila* T6Es are broad spectrum or generalists (active in synergy or targeting multiple species) or restricted-spectrum or specialists (active on a limited number of targets). A combination of several T6E efficiently targets a broad spectrum of bacterial strains (top), whereas V2b together with V5 seem to be sufficient to antagonize multiple bacterial taxa (medium spectrum); the three amidases V2a, V2b, and V5 separately target closely related strains, such as other family members *Stenotrophomonas* strains, *Xanthomonas* strains, and *P. fluorescens* or similar niche competitors like *P. agglomerans* strains; V6 seems to specifically target Gram-positive bacteria, such as *Microbacteriaceae*; P5 could be induced on solid medium or upon physical aggression and thus specifically target armed competitors (bottom).

In this study, we unveiled multiple T6SS features that explain the competitive efficacy and broad target of *S. rhizophila* on seed-borne bacteria. We described that *S. rhizophila* CFBP13503 carries a large T6E-I repertoire associated with only one T6SS, similarly to *Acidovorax citrulli* (13 T6Es) (59), *Serratia marcescens* (18 T6Es) (60), *Pseudomonas fluorescens* (20 T6Es) (61), and *Burkholderia cenocepacia* (29). The 13 *Sr*-T6Es display several putative activities, such as phospholipases, DNases, amidases, NADase, pore-forming, and metalloproteinases. The role for large effector repertoire acquisition is proposed to be a bet-hedging strategy for bacteria to minimize T6SS-resistance appearance and maximize T6SS potency in changing environments (30, 62, 63). A diversified repertoire of effectors should also allow to multiply enzymatic activities and combine antibacterial effects, leading to a fast cell death of the target (64). Here, we demonstrated that a large T6E repertoire notably allows to expand the target range by dedicated and specific-targeting effectors. Therefore, we refer to specific or narrow-spectrum and broad-spectrum effectors, acting as specific to a target or included in a synergistic antibacterial effect (Fig. 7). Broad-spectrum effectors include V3, P1, and P2, for which the deletion did not impact the T6SS activity in any target strain and are involved in the broad range competition. Specific effectors are the others involved in specific functions, such as intra-genus competition, defense against aggressive competitors, or adaptation to changing environments. We hypothesize that specific and broad-spectrum T6SS effectors could contribute to reinforce or partly explain the specialist or generalist strategy of competitors in the capture of external resources delivered by the attacked target strains (65). For Sr-CFBP13503, we argue that the association of different effector types provides a strong competitiveness in diverse and variable microbiomes, at least in seed-microbiomes.

The observation of variable GC contents from each effector (e.g., V3, V4, V6, V7, P1, P4, P5) and of their presence in several but limited bacterial families indicates a dynamic evolution and acquisition events from very distinct organisms, underlying a high specificity and a potential role in niche adaptation. Acquiring new T6SS effectors allows bacteria to outcompete their competitors by enhancing their arsenal of toxic proteins. This can lead to the replacement of ancestral effectors while retaining immunity genes to protect against kin bacteria that have not replaced the old effectors (66). The dynamic acquisition and deployment of T6SS effectors through HGT drive successive rounds of weapon optimization and selective sweeps, shaping the composition and structure of microbial communities (67). For example, *S. rhizophila* CFBP13503 and CFBP13529, which have a similar T6E-I repertoire, were co-isolated from radish seed and found to co-exist without harmful interactions (2). Inversely, the very close strain *S. rhizophila* CFBP9006, or the more distant and susceptible CFBP13524 and CFBP13525 strains, were isolated from a different host (bean, rapeseed) (9), supporting the hypothesis of a T6SS-based kin exclusion as observed in *Myxococcus xanthus* (68). The way of acquisition and stability of T6E-I pairs through HGT should be investigated for *S. rhizophila* in the seed and seedling microbiota environment.

Interestingly, we found the two fast-effective lytic amidases as the most potent *Sr*-T6Es. Lytic effectors have been described as an adaptive strategy to overcome the constraint of target availability due to dead cell physical barriers upon T6SS attacks (23). In this study, we showed that V2b and V5 are responsible for the major bursting cell death phenotype of target strains, notably happening quickly in the early competition. Peptidoglycan targeting effectors often possess a PG binding domain (LysM, SH3), which can serve as a specific binding site for specific PG architectures, and a catalytic domain allowing for hydrolysis of specific molecular links (M73, lysozyme, amidase). This structure suggests that amidases are specific to a target strain, which we observed with V2a, V2b, and V5, either specifically targeting closely related strains or synergistically targeting a broad but not wide spectrum. Amidases have been observed to be blocked by a natural peptidoglycan structure modification, increasing fitness when exposed to bacteriophages or antibacterial weapons (69). This suggests that the mix of diverse amidases with different functions should increase antibacterial potency against amidase-resistant peptidoglycans, which we observed with the combined activity of V2b and V5. These peptidoglycan modifications are accompanied by an important fitness cost (69), thus potentially explaining the weak distribution of V2a, V2b, and V5-associated immunities in seed-associated bacteria. T6SS amidases have evolved to carry dual function in a single protein, thus increasing antimicrobial efficiency (43, 70). *Acinetobacter baumannii* Tse4 bifunctional effector carries both transglycosylase and endopeptidase activities, allowing Gram-negative and Gram-positive targeting (43). V5 seems to be a dual-function amidase, but its activity and its role in targeting Gram-positive bacteria remain to be determined.

We report that *S. rhizophila* uses a polar, short, and highly dynamic T6SS to lyse target strains. This dynamic is comparable to what is observed in *A. baylyi* (35) and *A. baumannii* (34). Although *Acinetobacter* does not possess TssJ, its T6SS architecture and gene composition are very close to *S. rhizophila* (34). These three strains possess a short TssA form likely associated with rapid contraction. Moreover, the observation that the *Acinetobacter* T6SSs assemble through the whole cell, whereas *Sr*-T6SS never assembles a long sheath, could be explained by specific proteins stabilizing the apparatus, yet to be determined (71). One could expect that such a high firing frequency should secrete a high T6E quantity in a short period of time, thus increasing T6SS potency.

We linked the *Sr*-T6E-I secretion and production to their transcriptional expression. We first observed that 7 out of 10 effector-containing operons tested are co-regulated with the T6SS *tssJ* core gene. This revealed that not all effectors and additional immunities are expressed, but are inhibited or regulated. Moreover, cell proteome analysis proved the production of three P5-associated immunities, while P5 was present at very low level and P*paar5* was inactive in liquid conditions but activated in solid conditions. This revealed the complex regulatory architecture of T6E-I operons with potential internal promoters that ensure immunity expression to prevent self and sibling intoxication (72, 73). Intriguingly, *vgrG1*, *vgrG7,* and *paar5-tde5* operons were not expressed in liquid culture, but P5 has been involved in the antibacterial activity, suggesting a regulatory control. The observation of regulated T6E-Is suggests that *S. rhizophila* finely selects a subset of potent effectors to optimize the T6SS activity against a determined diversity of target strains and can adapt the repertoire in changing environments. It is the case in *Pseudomonas fluorescens* that upregulates *vgrG* genes in necrotic roots compared to healthy roots (74). Future work should determine the regulation and the expression variation of effectors within microbiota transmitted from seed to seedling (9). Our work sheds light on the importance of T6Es to target specific strains in microbial communities. From the perspective of microbiome engineering with efficient bacterial synthetic communities as seed inoculum against phytopathogens (75), it would be important to associate such a broad, effective, and competitive *S. rhizophila* strain and bacterial strains resistant to its T6SS.

## MATERIALS AND METHODS

### Bacterial strains and growth conditions

Strains used in this study are listed in Table S2. *Escherichia coli* DH5a was grown at 37°C in the Luria-Bertani medium (LB, Thermo Fisher Scientific, USA). All other strains, including *S. rhizophila,* were grown at 28°C in rich tryptic soy broth (TSB100, Thermo Fisher Scientific). When necessary, this medium was 10-fold diluted to produce less rich conditions (TSB10) or complemented with agar 14 g/L. Media were supplemented with appropriate antibiotics and inducers at the following concentrations: tetracycline 20 μg/mL, rifampicin 10 μg/mL, IPTG 10 μM (Sigma, USA).

### Proteomic analysis

For proteomic analysis of the total proteome and exoproteome (i.e., secretome), *S. rhizophila* CFBP13503 WT or Δ*hcp* cells were cultured in 50 mL TSB10 at 28°C for 17 h. Stationary phase cells were adjusted to an OD_600_ equal to 1 and 10-fold diluted in 50 mL of fresh TSB10 for a 5 h exponential culture. For each culture, 5 mL of OD_600_-adjusted aliquot (approximately 30 mg dry weight) was centrifuged at 5,000 × *g* for 2 min, and the supernatant was separated from the cell pellet. For total protein extraction, cell lysis was performed by bead beating and ultimately treated as described previously (76). For total cell proteome analysis, sample preparation and mass-spectrometry processing were performed as described previously (76, 77), using an Exploris 480 tandem mass spectrometer operated in data-dependent acquisition mode. Four biological replicates were used for each condition (Table S8). The mass spectrometry proteomics data have been deposited to the ProteomeXchange Consortium via the PRIDE partner repository with the dataset identifier PXD074895 and 10.6019/PXD074895.

### Plasmid construction

Primers used in this study are listed in Table S3. PCR amplifications were conducted using the Q5 High-Fidelity PCR Kit (NEB, USA) following the manufacturer’s protocol with incubation in the Bio-Rad T-100 Thermocycler (USA). PCR products were then purified using the NucleoSpin Gel and PCR Clean-up kit (Macherey-Nagel, USA) and stored at −20°C.

The plasmid pME6031Tc (78) was used as a vector for transcriptional fusion and cloned by the restriction/ligation method using restriction enzymes from NEB using the Quick Ligation Kit (NEB) according to the manufacturer’s instructions.

The plasmid pEX18Tc (79) was used to produce deletions of T6Es and insertion of *tssB*::sfGFP by the allelic exchange method as described previously (33). Cloning consisted of fusing 500–1,000 bp DNA fragments of upstream and downstream regions from the targeted sites using the HiFi DNA Assembly kit (NEB).

Chemically competent *E. coli* DH5α cells (100 μL) were transformed with 10 μL of the ligation reaction mix or 5 µL of the HiFi reaction mix using the heat shock protocol.

### Construction of *S. rhizophila* CFBP13503 strains and target strains

Deletion and insertion mutants were obtained by an allelic exchange method using pEX18Tc (33). CFBP13503 electrocompetent cells were transformed via electroporation (2 kV, 5 ms) with 100–200 ng of plasmid and selected as described in the supplementary information. Construction of strains with the pME plasmid was performed with the same procedure. Strains for fluorescence microscopy imaging were constructed by transformation of pME-P*lac-mCherry*. Basically, electrocompetent *P. fluorescens*, *P. agglomerans,* and *E. perscicina* cells were mixed with 500 ng of plasmid and transformed by electroporation as described above.

### Fluorescent reporter assays

Fluorescence assay was used to evaluate the predicted promoter activity of *vgrG* and *paar-tse* genes. Overnight cultures of CFBP13503 strains carrying pME-transcriptional fusion plasmids or the empty pME-*mCherry* plasmid were adjusted to an OD_600_ of 1, and 200 µL was dispensed into a 96-well black clear-bottom microplate (Greiner Bio-One, Austria) for fluorescence (F_570_, excitation: 520 nm, emission: 570 nm) and OD_600_ quantification with the VANTAstar microplate reader (BMG Labtech, Germany). To evaluate *mCherry* expression during the exponential phase, overnight cultures were adjusted to an OD_600_ of 0.1 in fresh TSB10 medium and incubated for 5 h at 28°C with agitation at 150 rpm. OD_600_ was then adjusted to 1 prior to measuring F_570_ and OD_600_ as described above. The experiment was performed in four biological replicates.

### Interbacterial competition assays

The impact of effector deletions on the T6SS antibacterial activity was measured by interbacterial competition assays using the qualitative lysis-associated β-galactosidase assay (LAGA) and the quantitative survivor growth kinetic (SGK) (80) or CFU plating methods when SGK was not achievable (9). LAGA is based on the degradation of the yellow chlorophenol red-β-D-galactopyranoside (CPRG) to a purple chlorophenol red by the target strain β-galactosidase released in the medium upon lysis. SGK is based on the time for a population to resume growth after a competition assay, which is proportional to the cell concentration and thus killing activity of the attacker. Briefly, attacker strains (CFBP13503 and derivatives) and target strains overnight cultures were adjusted to an OD_600_ of 1 and 2, respectively, and mixed in an equal volume. Ten microliters of the mixture was spotted onto TSA10 plates and incubated at 28°C for 4 h for *E. coli* or 6 h. LAGA was achieved by the addition of 10 μL of 2 mM CPRG (Sigma) on the spot. SGK was performed by the resuspension of each co-culture spot in 1 mL TSB100+Rif50 to select surviving target cells. One hundred microliters of each resuspension was added to 100 µL of TSB100+Rif50 in a 96-well microplate and incubated in the VANTastar microplate reader. CFU counting was performed by plating several dilutions of the resuspended spot and counting the emerging colonies on a selective medium. Experiments were performed in three or four biological replicates.

### Fluorescence microscopy

To determine the target cell death phenotype, attacker CFBP13503 WT and target strains carrying a constitutive mCherry or GFP expression were grown overnight in TSB10. Cultures were washed with TSB10, adjusted to an OD_600_ of 1, mixed at a 1:1 attacker: target ratio, and 1 µL was spotted onto a microscope slide poured with a 2% agarose pad (NuSieve, BMA) in a gene frame and covered with a coverslip. Cells were observed by fluorescence microscopy using a Zeiss Axio Imager Z2 microscope equipped with an Axiocam 305 color camera, at iMAC (IRHS cellular imaging platform). GFP and mCherry fluorescence were captured using 20% light and 100 ms exposure time. The competition was captured using time-lapse imaging of three to five different fields with 5 min intervals over a 1-h period. Image analysis consisted of tracking every cell death event using Fiji (81).

### Promoter prediction

Potential promoter sequences of *vgrG* and *paar-tse* clusters were predicted by the tool Promotech (82) running under a Python environment. To do so, the entire operon, as well as 5,000 bp directly upstream of *vgrG* or *paar-tse* genes, was submitted to Promotech analysis. The resulting predicted sequences were subjected to BPROM analysis (Softberry) (83) to identify putative regulatory sequences, such as −10 and −35 sites (Fig. 1).

### Protein characterization by predictive tools

To gain insight into the function of *vgrG* and *paar-tse* clusters, we used several online tools for protein characterization and interaction modeling that we describe in the supplementary information. Notably, AlphaFold3 was used to predict protein structure and protein-protein interactions (37). The potential interactions between proteins were considered positive for an ipTM score >0.75.

### Defense mechanism searching

*Sr*-T6E-I orthologous repertoire in 178 seed-associated strains (Table S9) was defined using a blastP with 30% identity and 30% length coverage threshold from *Sr*-T6E-I as a query. Hit sequences were processed with SignalP (84) to remove potential signal peptides for further analysis. The resulting hit table was manually curated for putative orthologous proteins. Sorting criteria were the automatic annotation similarity with the query, a signal peptide presence/absence compared to the query, and the alignment of the predicted structure with the query when necessary. The inhibitory activity of the orthologous *Sr*-T6I against the cognate *Sr*-T6E was predicted using AlphaFold3 with the *Sr*-T6E-I pair as a positive control. An ipTM score >0.75 was counted as an interaction.

Capsular genes in the 178 seed-associated strains were detected using CapsuleFinder (56) with both monoderm and diderm modes. Positive hits for monoderm capsular gene in Gram-negative and for diderm in Gram-positive bacteria were discarded.

### Seed-bacteria phylogeny and associated features

A phylogenetic tree of the 178 seed-associated bacterial strains was generated with Bakta annotated genomes (85) using GToTree analysis (86) with the following options: -H Bacteria -T IQ-TREE -B 1000. Basically, the “Bacteria” mode, which is based on 74 marker genes, was used and bootstrapped 1,000 times to draw the most confident tree. Genomic features determined as described above were inferred as a presence/absence matrix. The resulting tree was edited using the iTOL v7 tool (87).

### Statistical analysis and data formatting

Numeric results from experiments were compared for statistical significance using the Wilcoxon or *t*-test analysis in R Studio. Schematics of biological models were produced using Biorender.

## SUPPLEMENTAL MATERIAL

Supplemental figures spectrum.03532-25-s0001.pdf Fig. S1 to S10.

Supplemental text spectrum.03532-25-s0002.docx Supplemental materials and methods.

Supplemental legends spectrum.03532-25-s0003.docx Legends for supplemental figures and tables.

Supplemental tables spectrum.03532-25-s0004.xlsx Tables S1 to S9.

Supplemental file spectrum.03532-25-s0005.pptx Time lapse of killing phenotypes of Sr against Xcc and Ec

ASM does not own the copyrights to Supplemental Material that may be linked to, or accessed through, an article. The authors have granted ASM a non-exclusive, world-wide license to publish the Supplemental Material files. Please contact the corresponding author directly for reuse.
