# Supplementary figures and images for: Specific and broad-spectrum antibacterial effectors of type VI secretion system drive competition of Stenotrophomonas rhizophila against bacteria from seed microbiota
Source: Microbiol Spectr. 2026 Jun 15;14(7):e03532-25. doi: 10.1128/spectrum.03532-25 (PMC13340143; doi:10.1128/spectrum.03532-25)

Figure S1

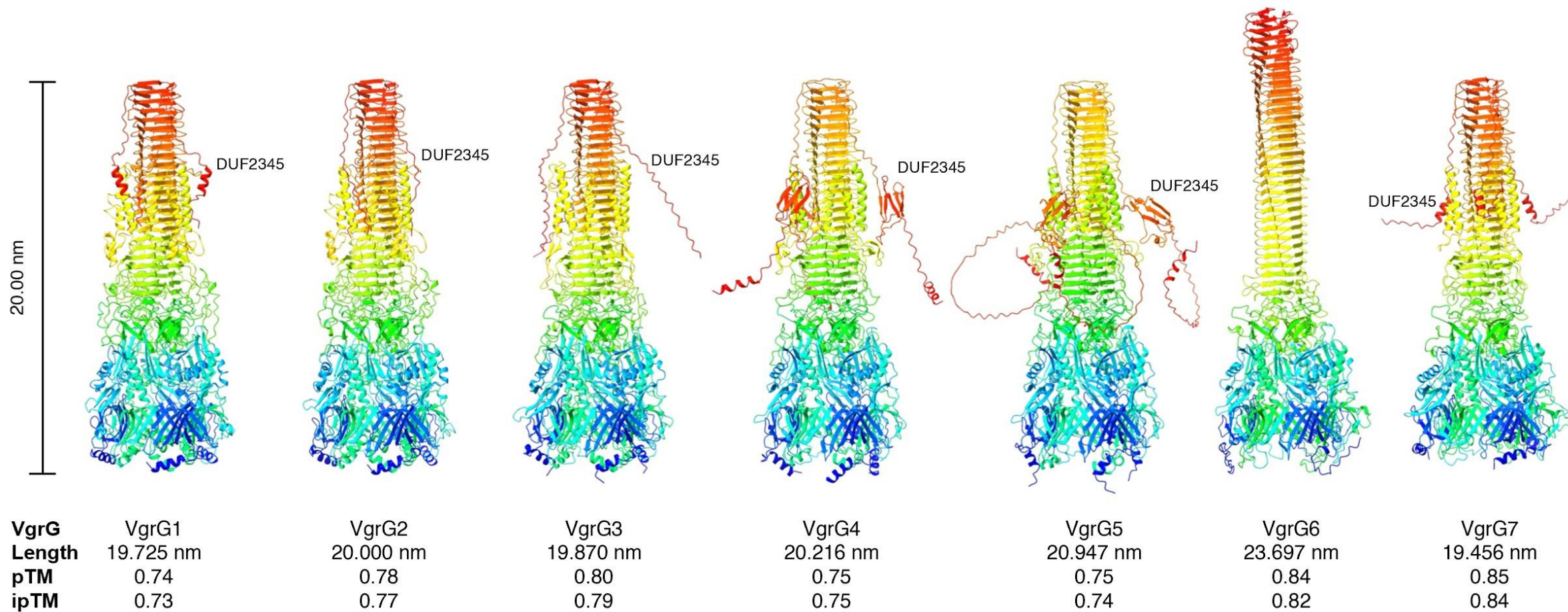

Figure S2

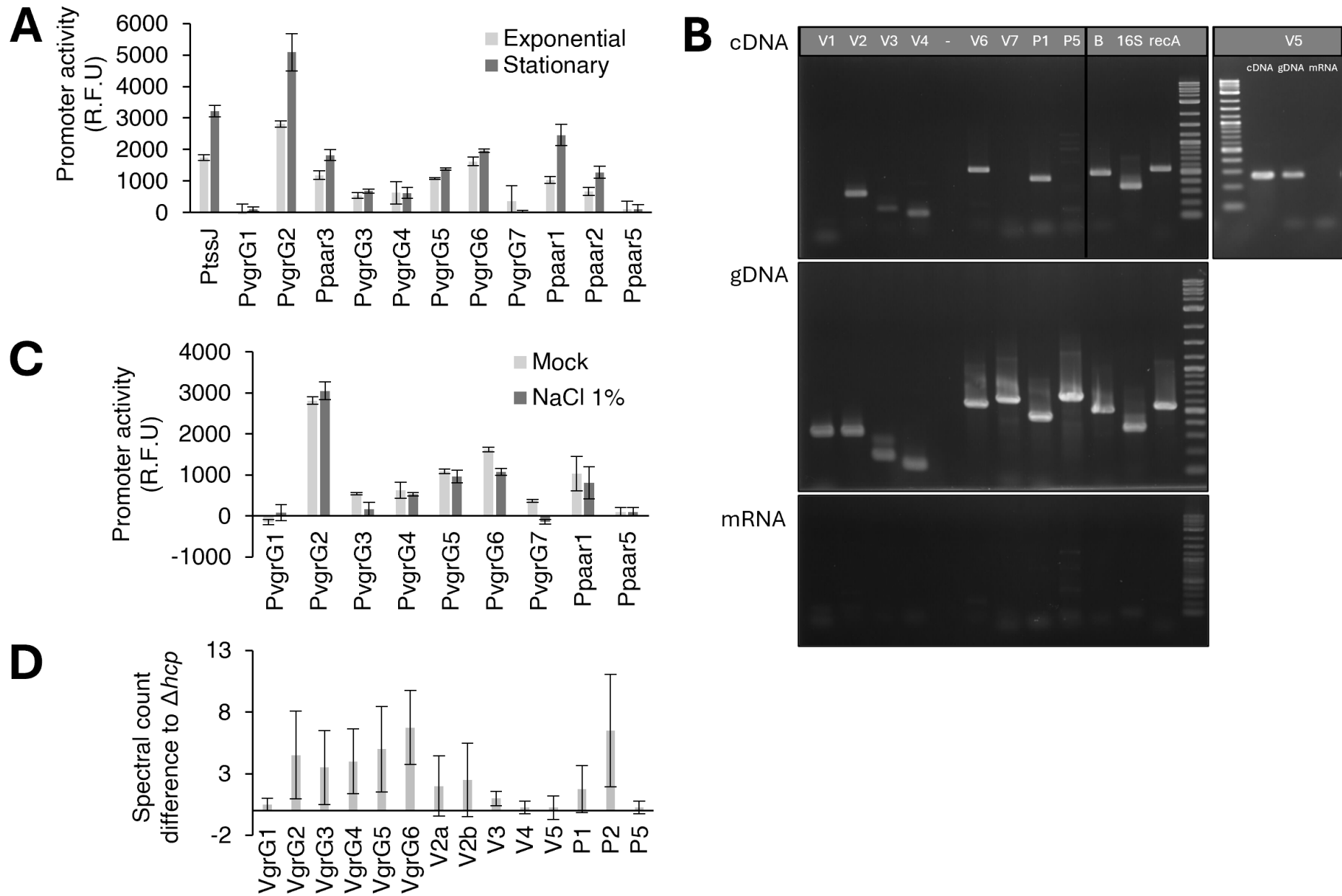

Figure S3

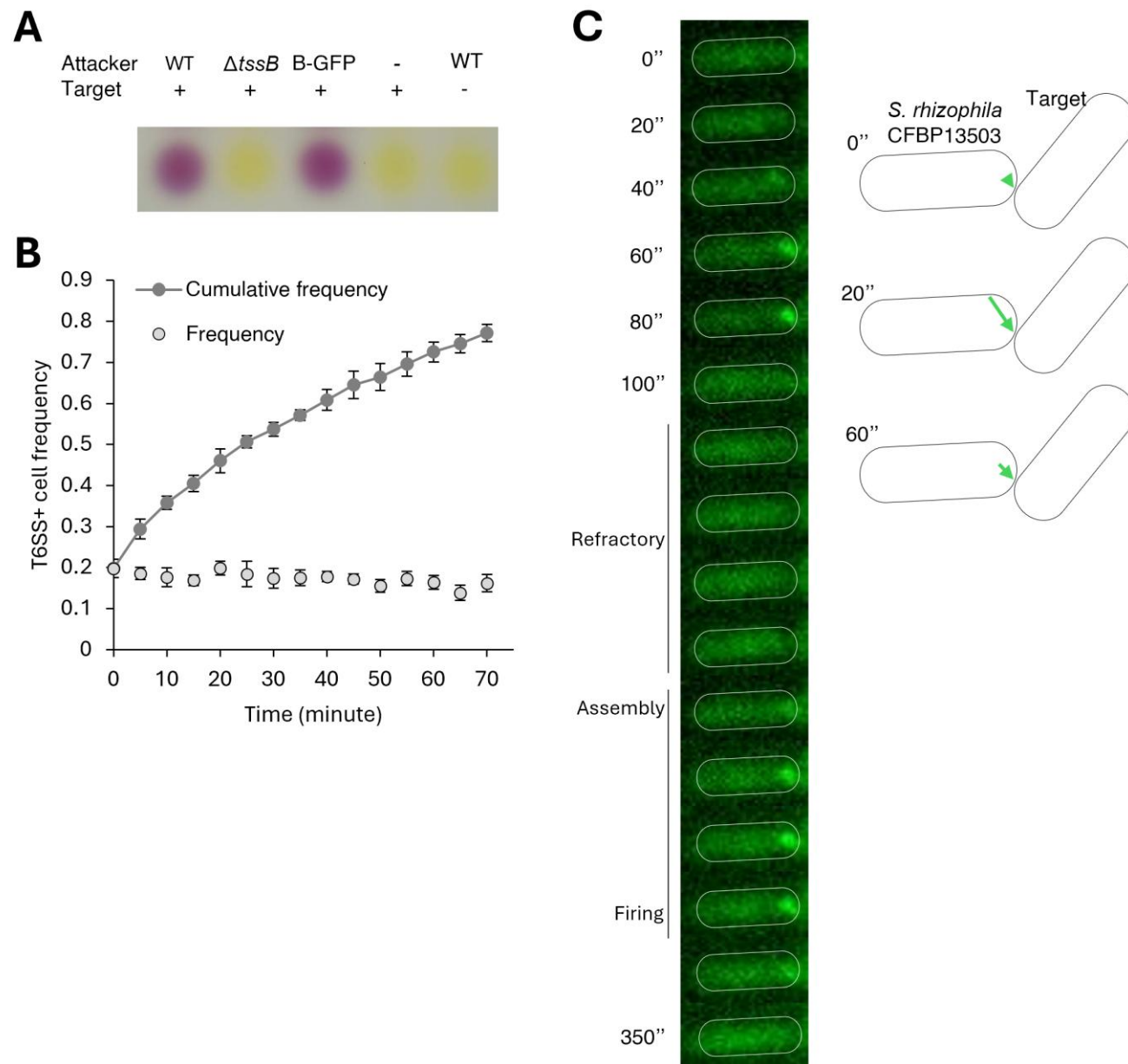

Figure S4

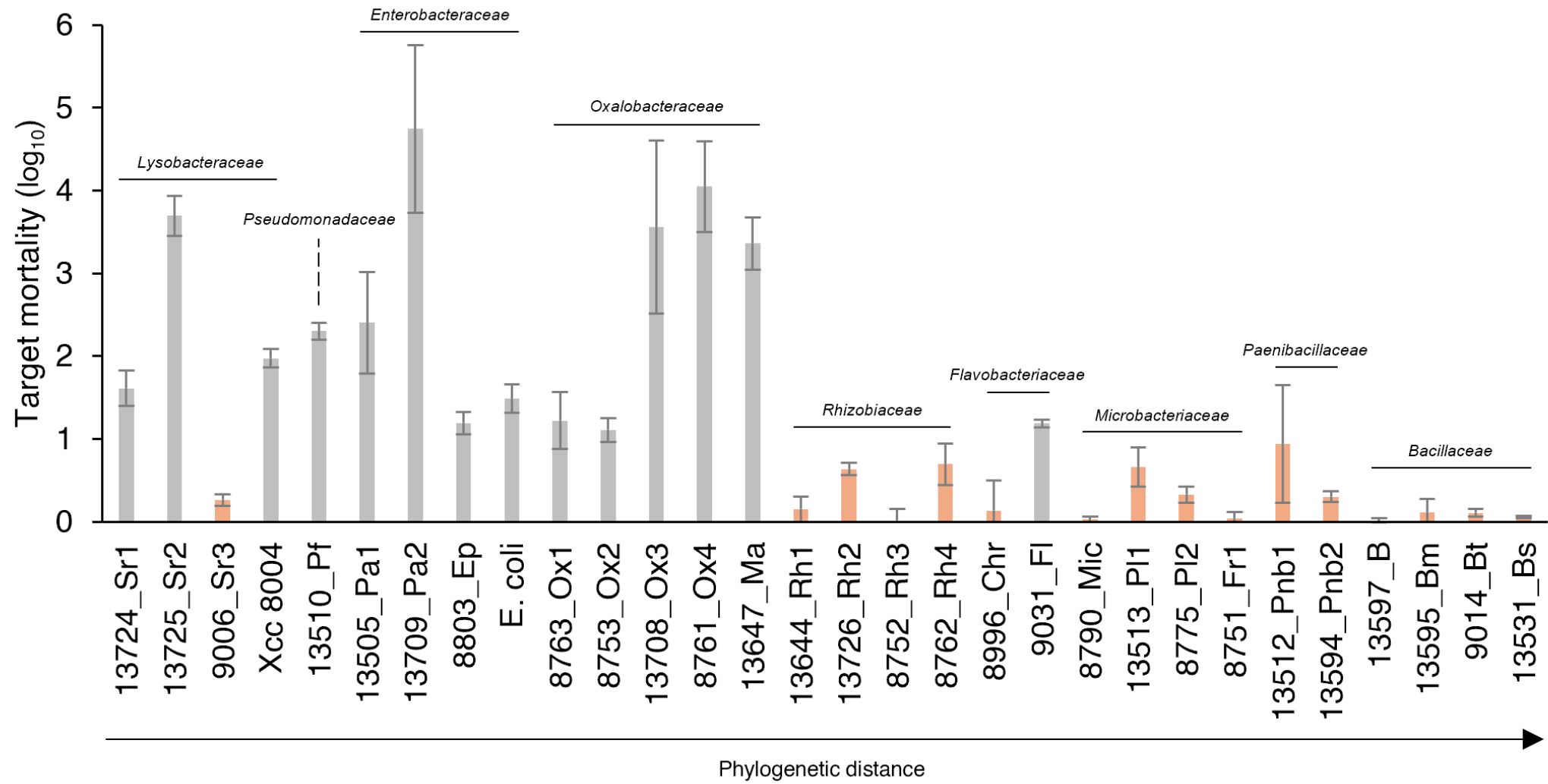

Figure S5

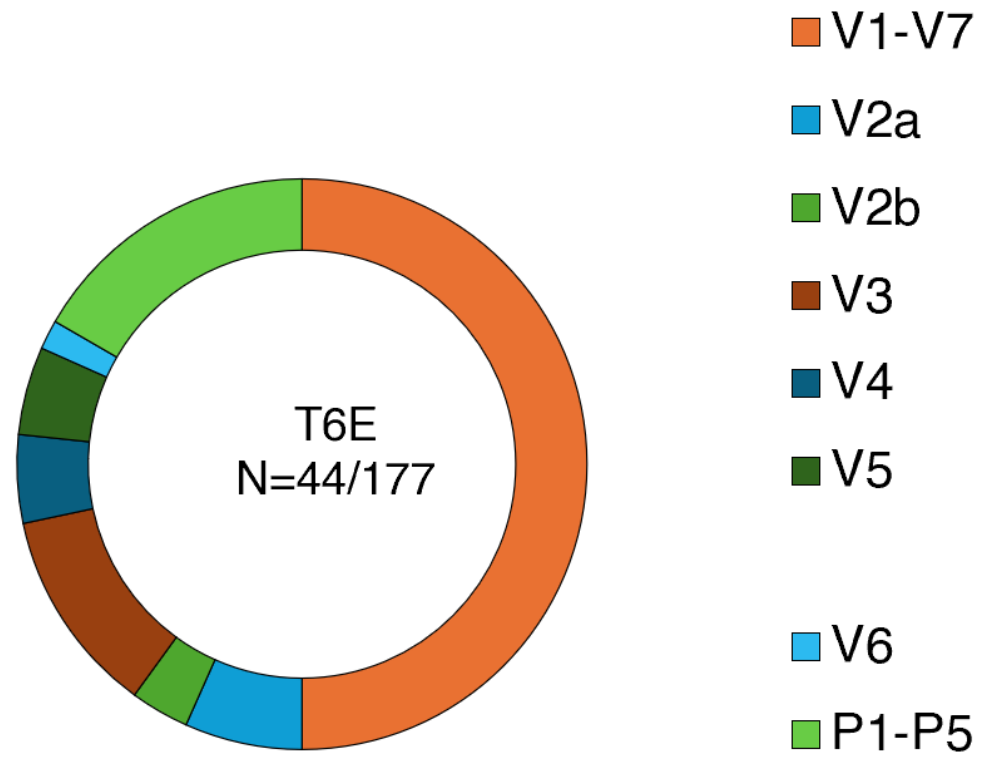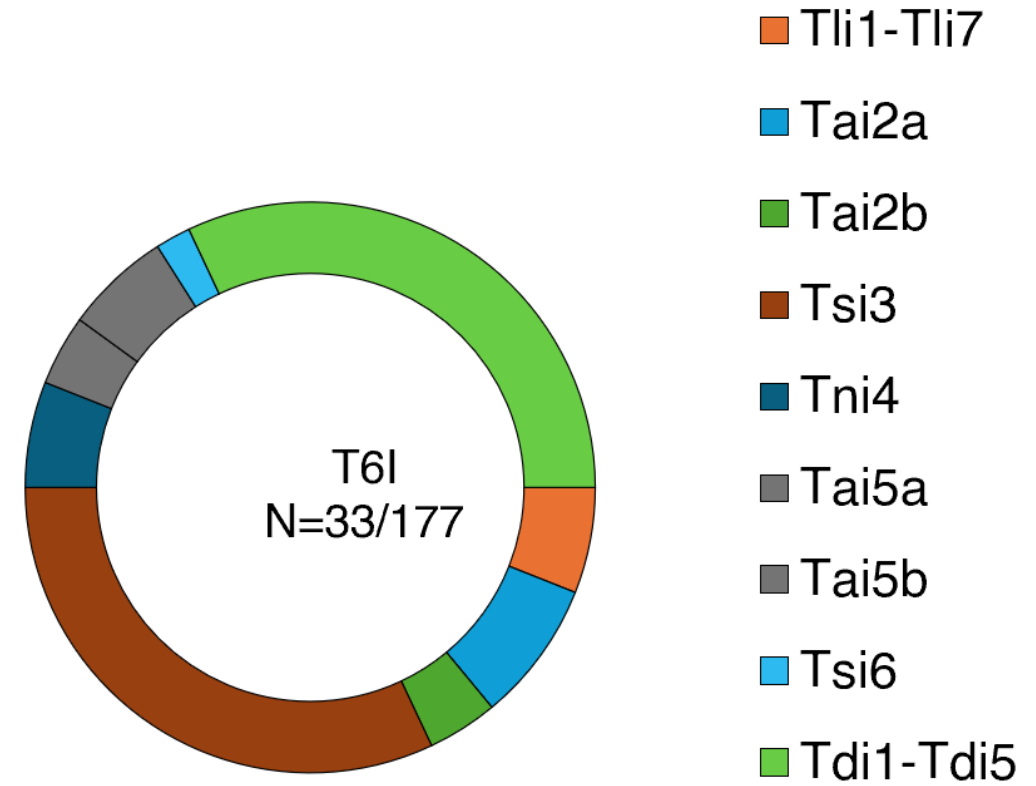

Figure S6

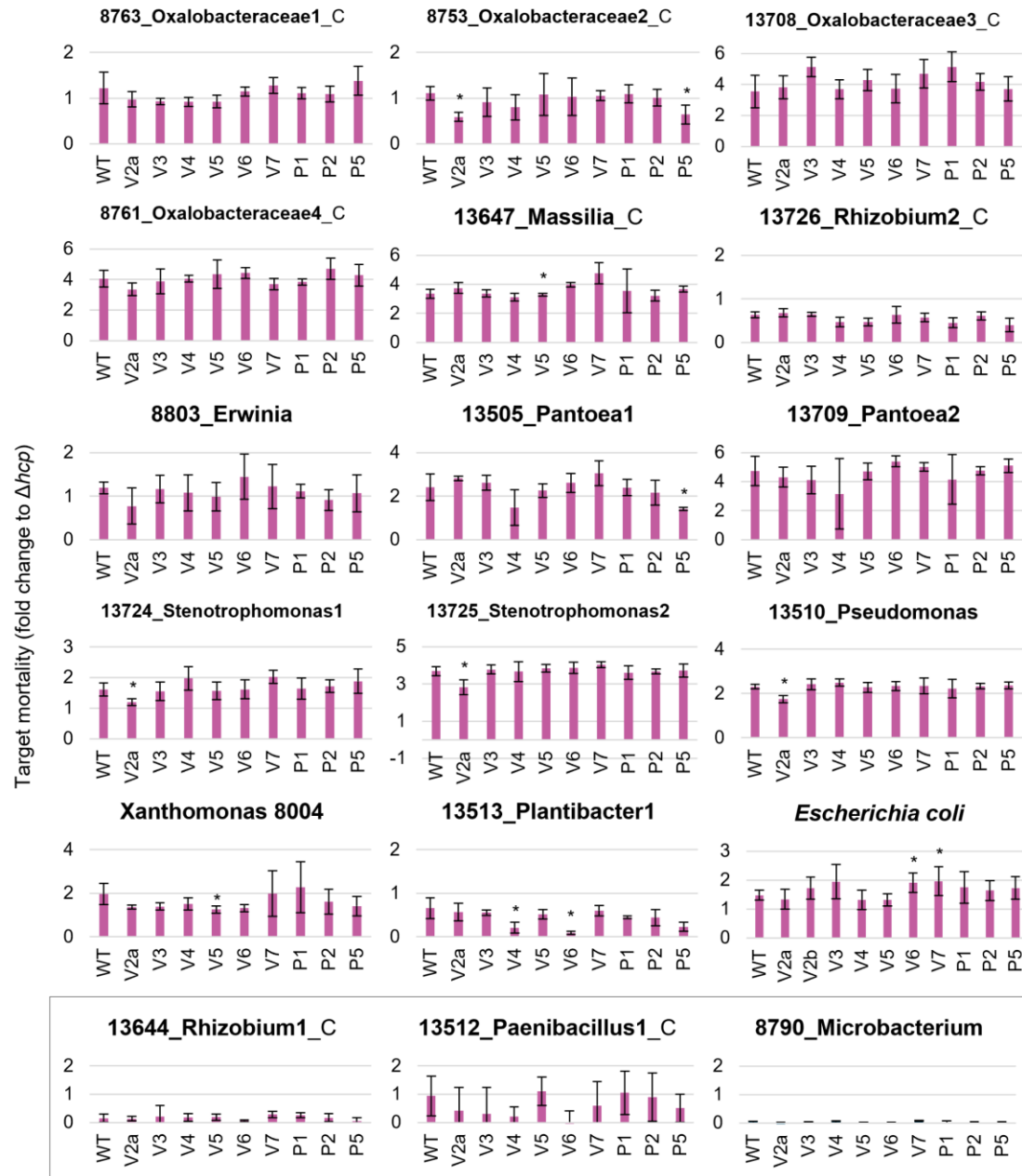

Figure S7

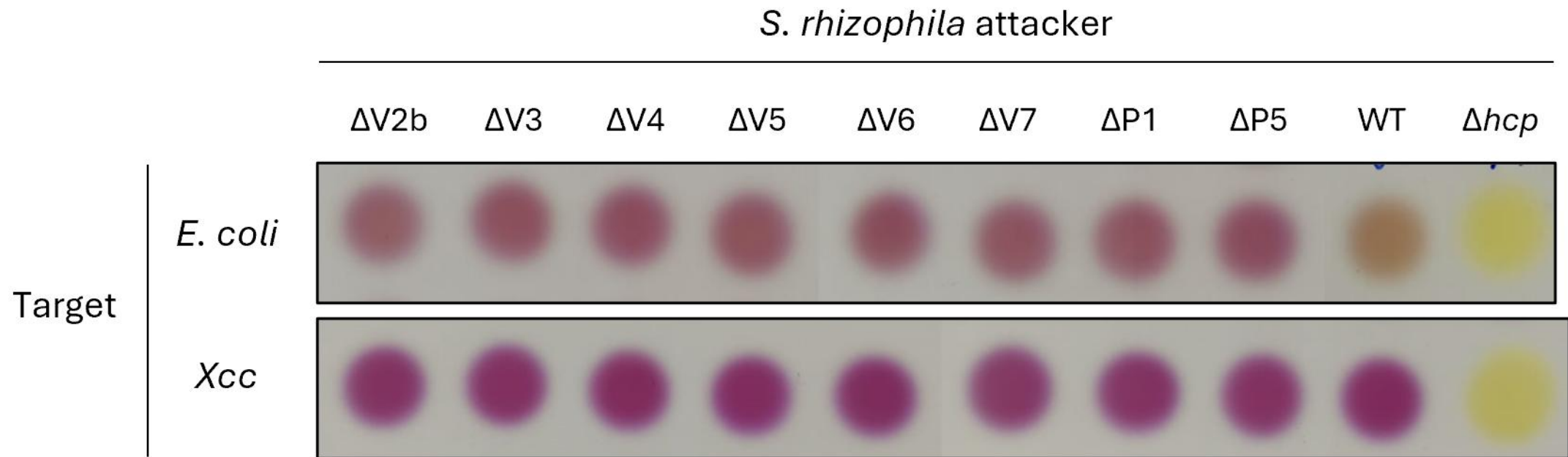

Figure S8

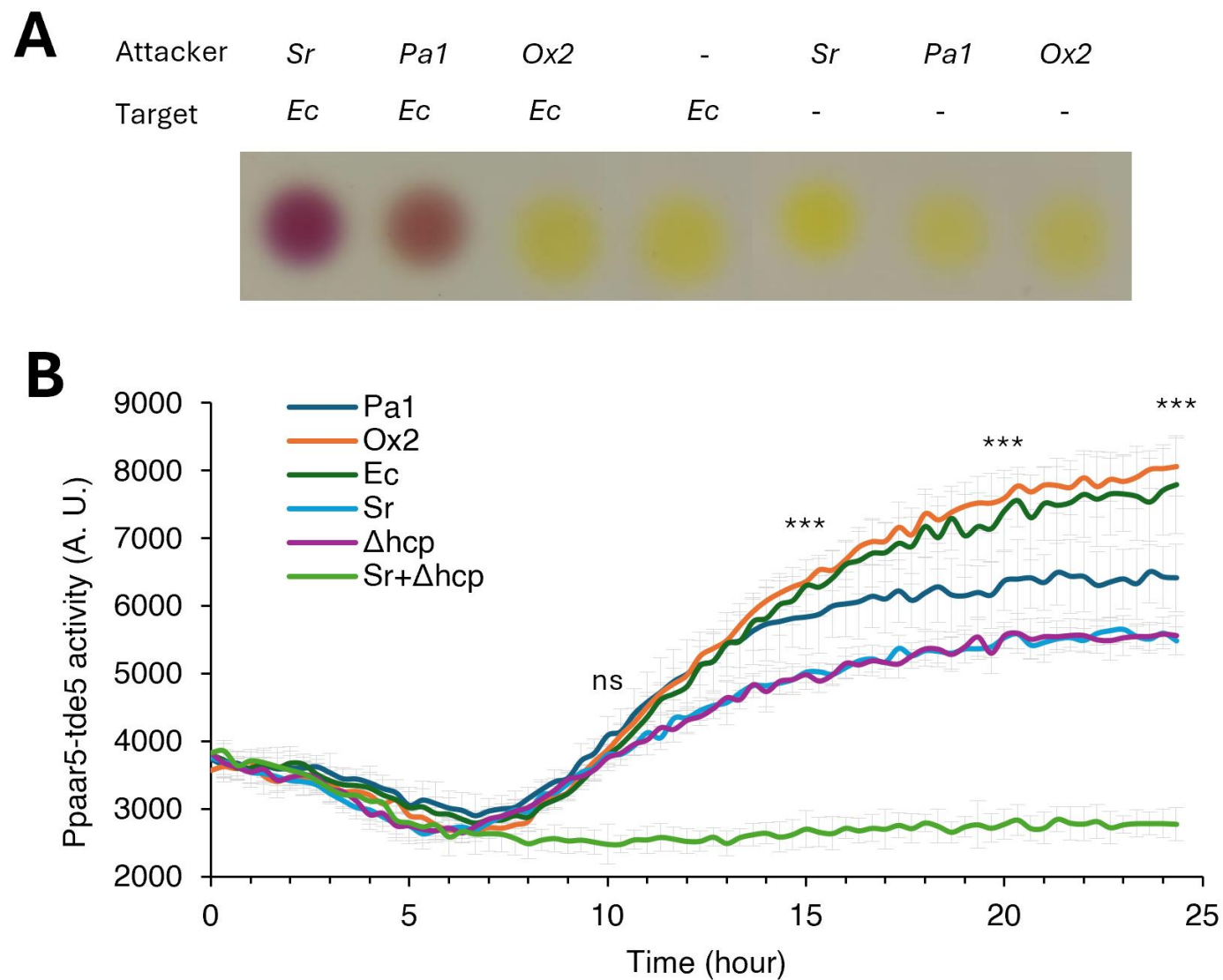

Figure S9

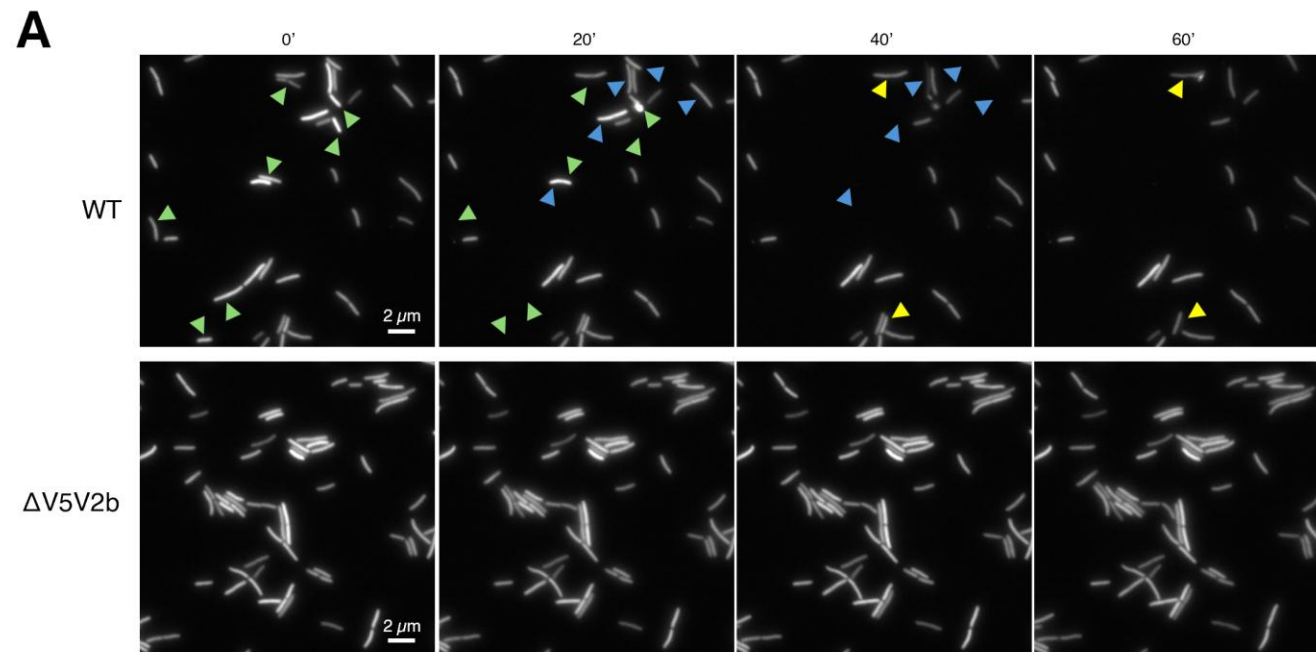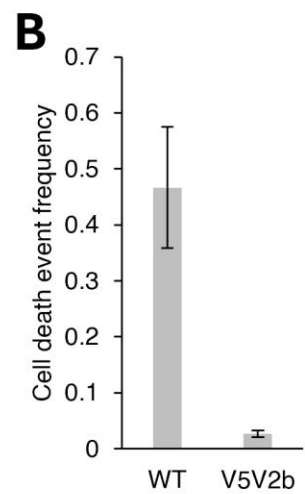

Figure S10

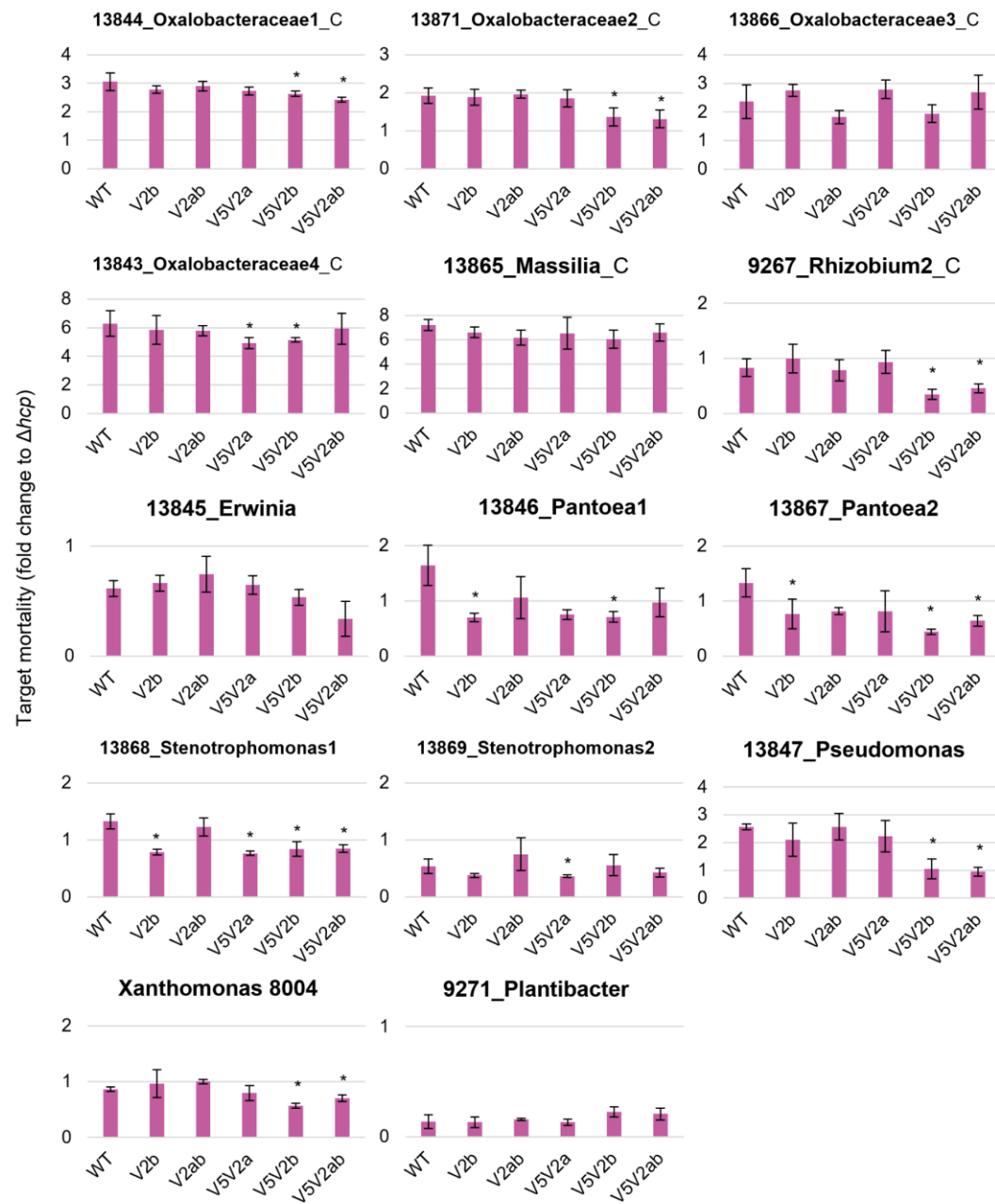

Supplement: Supplemental figures — Fig. S1 to S10. [file spectrum.03532-25-s0001.pdf]
