# Supplementary material for: Specific and broad-spectrum antibacterial effectors of type VI secretion system drive competition of Stenotrophomonas rhizophila against bacteria from seed microbiota
Source: Microbiol Spectr. 2026 Jun 15;14(7):e03532-25. doi: 10.1128/spectrum.03532-25 (PMC13340143; doi:10.1128/spectrum.03532-25)
Supplement: Supplemental text — Supplemental materials and methods. [file spectrum.03532-25-s0002.docx]

**SUPPLEMENTAL MATERIAL AND METHODS**

**Growth conditions**

Strains used in this study are listed in Table S1. *Escherichia coli* DH5a was grown at 37°C in Luria-Bertani (LB, Thermo Fischer scientific, USA) medium (10 g/L tryptone, 5 g/L yeast extract, 10 g/L NaCl). All other strains, including *S. rhizophila* were grown at 28°C in rich tryptic soy broth (TSB100; 17 g/L tryptone, 3 g/L soybean peptone, 2.5 g/L glucose, 5 g/L NaCl, 5 g/L K2HPO4, Difco) and on tryptic soy agar plates (TSA100: TSB100 medium with 14 g/L agar, Difco). When necessary, these media were diluted ten-fold to produce lesser rich conditions (TSB10 and TSA10). Liquid cultures were incubated with agitation at 150 rpm. Media were supplemented with appropriate antibiotics and inducers at the following concentrations: tetracycline 20 μg/mL, rifampicin 10 μg/mL, IPTG 10 μM (Sigma).

**RNA extraction**

To evaluate the transcription of *vgrG* and orphan *paar* genes, RNA extraction was performed using a phenol-chloroform protocol (1). *S. rhizophila* CFBP13503 was grown overnight in TSB10 at 28°C with agitation. Cultures were diluted in fresh TSB10 and harvested after 5h of incubation in the same conditions. Cells were adjusted to OD_600_ 0.5 for mRNA extraction by phenol/chloroform method. Basically, 1 mL of cell suspension was centrifugated and the supernatant was discarded. Cell pellet was resuspended in 1 mL of pre-warmed TRIzol^TM^ (Invitrogen, USA) and incubated 5 min at 65°C. Then, 200 µL of cold chloroform was mixed, vortexed and incubated on ice for 2 min. The preparation was centrifugated at 12,000 rpm for 15 min at 4°C and the upper phase (approximatively 500 µL) was transferred to a new RNase-free tube containing 400 µL of cold isopropanol. The mixture was incubated 10 min at 15°C and centrifugated at 12,000 rpm for 20 min at 4°C. The supernatant was discarded, and the precipitated pellet was washed twice with 1 mL of cold ethanol 70% by centrifugation 12,000 rpm for 10 min at 4°C. The pellet was finally air dried for 10 min at room temperature re-hydrated with 70 µL nuclease free water. RNA concentration was measured with a NanoDrop One (Thermo Fisher, USA) and samples were stored at -20°C for rapid use or at -80°C. DNA contamination was then removed using the TURBO DNA-free kit (Ambion, USA), following the manufacturer's instructions. DNase-I-treated samples were purified with the phenol/chloroform method as described below. Briefly, DNase I-treated samples were mixed to 1 volume of TRIzol^TM^, centrifugated and selected for upper phase. Next, 1 volume of chloroform was added, centrifugated and selected for upper phase. Then, 2 volumes of ethanol 100% was added and the mixture was incubated at -20°C for 1h. Finally, the mixture was centrifugated and air dried to remove residual ethanol. mRNA pellet was finally re-hydrated with 20 µL of RNase-free water and stored at -80°C.

**RNA Retrotranscription**

For further applications, mRNAs were reverse transcribed into cDNAs with random primers using the ProtoScript® II First Strand cDNA Synthesis Kit (NEB) according to manufacturer instructions.

**RT-PCR**

cDNAs were then used to amplify *vgrG* and *paar* as well as *tssB*, *recA*, and 16S rRNA genes as positive controls of mRNA expression. At amplification cycles 30, 35, and 40, 5 μL of each reaction mixture were loaded onto a 1.5% agarose TAE gel and electrophoresed at 100V for 30 minutes. Control PCRs were also performed on genomic DNA as a positive control for primer annealing and on extracted RNAs as a negative control for DNA contamination.

**Proteomic analysis**

For proteomic analysis of total proteomes and exoproteomes (secretome), *S. rhizophila* CFBP13503 WT or Δ*hcp* cells were cultured in 50 mL TSB10 at 28°C for 17h. Stationary phase cells were adjusted at an OD_600_ equal to 1 and ten-fold diluted in 50 mL of fresh TSB10 for 5h exponential culture. For stationary and exponential phase cultures, 5mL of OD_600_-adjusted aliquot (approximatively 30 mg dry weight) was centrifugated at 5, 000 x *g* for 2 minutes and the supernatant was removed. For total protein extraction, cell pellets were resuspended in 60 µL LDS 1X buffer (Sigma) per mg of biomass and heated at 98°C for 5 min before sonication in a ultrasonic bath. Cell lysis was performed by bead beating (2) and the supernatant was centrifugated 16,000 x *g* for 1 min, transferred to a new Eppendorf tube before incubation at 98°C for 5 min and ultimately conserved at -20°C. The culture supernatants were treated with EDTA 1 mM and centrifugated at 5,000 x *g* for 10 minutes. Supernatants were transferred in a new falcon tube and centrifugated at 10,000 x *g* for 10 min at 4°C and repeated once. Samples were then treated with 10% trichloroacetic acid (TCA, Sigma) for protein precipitation and incubated 30 min on ice. A last centrifugation at 10,000 x *g* for 10 minutes was performed prior to sample conservation at -80°C. For total cell proteome or secretome analysis, sample preparation and mass-spectrometry processing were performed as described previously (2,3). Four biological replicates were used for each condition.

**Preparation of chemically competent and electro-competent cells**

Chemically competent *E. coli* DH5α cells were prepared as follow: the strain was plated on LB and grown overnight at 37°C. Several colonies were resuspended in 2 mL LB for overnight incubation at 37°C with agitation at 150 rpm. The culture was then 100-fold diluted in 10 mL fresh LB and incubated until exponential phase at 37°C with agitation. Exponential phase cells were placed on ice for 5 minutes and centrifugated at 5,000 x g for 5 minutes at 4°C. Supernatant was discarded, cells were resuspended in CaCl_2_ 0.1 M and chilled on ice for 1h. Cells were centrifugated and washed with glycerol 10 % with another round of centrifugation. Finally, cells were resuspended in 500 µL glycerol 10 % and 100 µL were aliquoted in a 1.5 mL Eppendorf tube prior to be stored at -80°C.

Electro-competent *S. rhizophila*, *P. fluorescens, P. agglomerans, E. perscicina and X. campestris pv. campestris* cells were prepared as follows: strains were plated on TSA100 and grown overnight at 28°C. Several colonies were resuspended in 2 mL TSB100 for overnight incubation at 28°C with agitation at 150 rpm. The cultures were then 10-fold diluted in 10 mL fresh TSB100 and incubated until exponential phase at 28°C with agitation. Exponential phase cells were placed on ice for 5 minutes and centrifugated at 5,000 x g for 5 minutes at 2°C. Supernatant was discarded and cells were washed three times with centrifugation at 5,000 x g for 10 minutes in ice-cold glycerol 10 %. Finally, cells were resuspended in 200 µL glycerol 10 % and 100 µL were aliquoted in a 1.5 mL Eppendorf tube prior to be stored at -80°C.

Electro-competent cells were transformed *via* electroporation (2 kV, 5 ms) with 100-200 ng of the suicide vector pEX18Tc. After electric shock, cells were immediately resuspended in 900 μL TSB100 and incubated for 2h at 28°C. Transformants were selected on TSA100 agar plates supplemented with tetracycline 20 µg/mL. Subsequently, several colonies were cultured in TSB100 medium at 28°C and 150 rpm for 3 hours prior to be spread onto TSA100 agar plates supplemented with 5% saccharose. Transformation with pME plasmids consisted in the same procedure with one selection in TSA100 supplemented with tetracycline 20 µg/mL. The resulting colonies were subjected to a PCR analysis and sequencing to confirm the deletion or detect the plasmid presence

**Plasmid construction and transformation**

**PCR amplification.** PCR amplifications were conducted using the Q5 High-Fidelity PCR Kit (NEB) following the manufacturer's protocol using the Bio-Rad T-100 Thermocycler. PCR products were then purified using the NucleoSpin Gel and PCR Clean-up kit (Macherey-Nagel).

**Cloning.** The plasmid pME6031Tc (4) was used as a vector for transcriptional fusion and. Cloning of promoters upstream the *mCherry* gene was performed the restriction/ligation method using restriction enzymes from NEB. First, we inserted m*Cherry* at KpnI and BglII sites to produce the pME-*mCherry*. Then, the 400 bp upstream regions from start codon of each *vgrG* and orphan *paar*­-*tse* genes were PCR-amplified from the genomic DNA of *S. rhizophila* CFBP13503 using specific primer pairs (**Table S1**). The amplified region thus excludes the start codon but includes RBS and potential regulatory sequences upstream the gene. After amplification, pME-*mCherry* and the DNA fragment were purified, digested with KpnI and HindIII, and cloned together using the Quick Ligation Kit (NEB) according to the manufacturer's instructions. Chemically competent *E. coli* DH5α cells (100 μL) were transformed with 10 μL of the ligation reaction mix using the heat shock protocol: the mixture was incubated at 42°C for 2 minutes, chilled on ice for 2 minutes and resuspended in 900μL of LB. This was followed by incubation at 37°C for 1 hour with agitation at 170 rpm. Afterward, the cells were spread onto LB agar plates containing tetracycline 10 µg/mL and incubated overnight at 37°C.

The plasmid pEX18Tc (5) was used to produce deletions of T6Es and insertion of *tssB*::sfGFP by the allelic exchange method. Cloning consisted of a DNA fragment assembly using the NEBuilder HiFi DNA Assembly kit (NEB) according to the manufacturer's instructions, where each primer used includes sequences that overlap each other for DNA assembly. Firstly, 500 to 1000 bp upstream and downstream the targeted sites were PCR-amplified from the genomic DNA of CFBP13503 using primer pairs carrying 20 bp homology sequences with the plasmid (Table S2). Second, the pEX18Tc vector was linearized by SacI digestion following the manufacturer's protocol. The linearized vector as well as PCR products were then purified as described above. Subsequently, ligation of the upstream and downstream flanking regions with the linearized vector was facilitated using the HiFi DNA Assembly kit (NEB). Chemically competent DH5α were transformed with 5μL of the HiFi reaction mix as described above. The plasmid for insertion of the translational reporter *tssB*::sfGFP was produced with 3 PCR fragments including the *tssB* sequence deleted for the STOP codon and carrying the *tssB* upstream region, the sfGFP deleted for the START codon, and the downstream region of *tssB*.

The transformed DH5α colonies were screened for plasmid detection by PCR. Positive clones for the constructed plasmid were cultured for plasmid extraction using the Nucleospin Plasmid Purification kit (Macherey-Nagel) according to the manufacturer's instructions. The plasmid construction was checked by Sanger sequencing in GeneWiz company.

**Fluorescent reporter assays**

Fluorescence assay was used to evaluate the predicted promoter activity of *vgrG* and *paar* genes. Cultures of CFBP13503 strains carrying pME-transcriptional fusion plasmids or the empty pME-mCherry plasmid were incubated overnight at 28°C with agitation at 170 rpm in 2 mL of TSB10 supplemented with the appropriate antibiotic. OD_600_ of overnight cultures was adjusted to 1 and 200 μL was dispensed into a 96-well black clear-bottom microplate (Greiner Bio-One, reference 675097). To evaluate *mCherry* expression during the exponential phase, overnight cultures were adjusted to an OD_600_ of 0.1 in fresh TSB10 medium and incubated for 4 hours at 28°C with agitation at 170 rpm. OD_600_ was then adjusted to 1 prior to measuring F_570_ and OD_600_ as described above. Fluorescence of the mCherry (F_570_, excitation: 520 nm, emission: 570 nm) and OD_600_ were quantified by endpoint measurements using the VANTAstar microplate reader (BMG Labtech). Fluorescence values were normalized to the corresponding OD_600_ values using the formula F_570_/OD_600_. Normalized fluorescence values were then subtracted from the empty vector values.

**Interbacterial competition assays**

The impact of effector deletions on the T6SS antibacterial activity was measured by interbacterial competition assays. The methodologies employed in this study are an adaptation of the qualitative Lysis-Associated β-Galactosidase Assay (LAGA) and the quantitative Survivor Growth Kinetic (SGK) protocols (6). Because some target strains are not subjected to SGK due to a slower growth rate in liquid culture, suspension plating was used to assess quantitative survival by CFU counting. Briefly, attacker strains (CFBP13503 and derivatives) and target strains (*E. coli* W3110 and other rifampicin resistant strains) were incubated overnight at 28°C in 2 mL of TSB10. Culture of *E. coli* was supplemented with 10 μM IPTG to induce β-galactosidase expression. Attacker and target strain cultures were adjusted to an OD_600_ of 1 and 2, respectively, in fresh TSB10 medium and mixed in an equal volume. 10 μL of the mixture was spotted onto TSA10 plates and incubated at 28°C for 4 hours for *E. coli* or 6 hours. The co-culture of the target strain with the T6SS-deficient strain of CFBP13503 Δ*hcp* was used as a negative control of antibacterial activity (7). Attackers and target strains alone were spotted as negative controls. The qualitative monitoring of the T6SS antibacterial activity (LAGA) was achieved by the addition of 10 μL of 2 mM chlorophenol-red-galactopyranoside (CPRG, Sigma) to one spot every hour. The target cell lysis is indicated by the color change of the spot due to CPRG hydrolysis (by β-galactosidase). The quantitative monitoring of the T6SS antibacterial activity (SGK) was performed by the resuspension of each co-culture spots in 1 mL TSB100 supplemented with rifampicin 50 µg/mL to select surviving target cells. Serial ten-fold dilutions of the Δ*hcp* condition were prepared to mimic log_10_ mortality changes. 100 μL of each resuspension was added to 100 µL of TSB100+Rif in a 96-well microplate (Greiner Bio-One, ref 655161). The microplate was incubated at 300 rpm and appropriate temperature for 24 to 48 hours in the VANTastar microplate reader. OD_600_ measurements were recorded every 5 minutes. Growth kinetic curves were analyzed to determine the time to reach approximatively half of the exponential growth phase (Tmid). Cultures with more surviving target cells (low T6SS activity) reached Tmid faster than those with fewer survivors (high T6SS activity). Tmid values from serial log-diluted Δ*hcp* condition were plotted to generate a linear regression curve, estimating log_10_ mortality change.

**Fluorescence microscopy**

The T6SS activity was also measured by fluorescence microscopy using a Zeiss Axio Imager Z2 microscope equipped with an Axiocam 305 color camera, at IRHS cellular imaging platform iMAC (https://www.sfrquasav-angers.org/plateaux-techniques/imac-imagerie-cellulaire). The filters included a 572/25 nm bandpass excitation filter, a 590 nm beamsplitter, and a 629/62 nm bandpass emission filter for capturing mCherry fluorescence, and a 470/40 nm bandpass excitation filter, a 495 nm beamsplitter, and a 525/50 nm bandpass emission filter for capturing GFP fluorescence. GFP and mCherry fluorescence were captured using 20% light and 100 ms exposure time. The T6SS dynamic and activity was measured with the B-GFP strain cultured in TSB10, centrifugated and concentrated ten-fold. One microliter of the sample were spotted onto a microscope slide poured with a 2% agarose pad (NuSieve, BMA) in a gene frame and covered with a coverslip. To determine the T6SS+ cell frequency, endpoint images of at least 3 different fields were taken and each B-GFP focus was manually counted using Fiji (ImageJ) software (Schindelin et al., 2012). To determine T6SS+ cell frequency and T6SS dynamic over time, a time lapse with 20 s or 5 min interval acquisition was performed. To determine the target cell death phenotype, attacker CFBP13503 WT and target strains carrying a constitutive mCherry or GFP expression were grown overnight in TSB10. Cultures were washed with TSB10, adjusted at OD_600_ of 1, mixed at 1:1 attacker:target ratio and 1 µL was spotted on agar pad as described above. The competition was captured using time-lapse imaging of 3 to 5 different fields with 5 minutes intervals over a 1-hour period. Image analysis consisted of tracking every cell death event using Fiji.

**Protein characterization by predictive tools**

To gain insight into the function of *vgrG* and *paar-tse* clusters, we used several online tools for protein characterization and interaction modelling. BlastP (NCBI) and Cblaster (CAGECAT) were used to find homologues and compare amino acid sequences between June and August 2025. CAGECAT online tool InterproScan was used to identify functional domains (8,9). SignalP-5.0 server (10) was used to predict signal peptides that were removed from the amino acid sequence for further analysis. TMMHM 2.0 sever (11) was used to predict transmembrane domains and protein localization. AlphaFold3 was used to predict protein structure and protein-protein interactions (12). The potential interactions between proteins were considered positive for a ipTM score > 0.75 (13,14). The resulting structures were subjected to DALI server (15) and FoldSeek (16) to identify the potential function and structural orthologues in the PDB (RCSB.org). Structural orthologues were superimposed with the query structure using the Matchmaker tool from ChimeraX software (17).

**References**

1. Peters, M.K., Astafyeva, Y., Han, Y., Macdonald, J.F.H., Indenbirken, D., Nakel, J., Virdi, S., Westhoff, G., Streit, W.R. and Krohn, I. (2023) Novel marine metalloprotease—new approaches for inhibition of biofilm formation of Stenotrophomonas maltophilia. *Appl Microbiol Biotechnol*, **107**, 7119–7134.

2. Lozano, C. and Armengaud, J. (2025) Sample Preparation and Processing for Quick, Universal, and Insightful Microbial Proteomics. In Islam Williams, T. (ed.), *Tissue Proteomics: Methods and Protocols*, Springer US, New York, NY, pp. 57–69.

3. Armengaud, J. and Duport, C. (2017) Chapter Eleven - Exoproteomics of Pathogens: Analysis of Toxins and Other Virulence Factors by Proteomics. In Shukla, A. K. (ed.), *Methods in Enzymology*, Proteomics in Biology, Part B, Academic Press, Vol. 586, pp. 211–227.

4. Wilton, R., Ahrendt, A.J., Shinde, S., Sholto-Douglas, D.J., Johnson, J.L., Brennan, M.B. and Kemner, K.M. (2018) A New Suite of Plasmid Vectors for Fluorescence-Based Imaging of Root Colonizing Pseudomonads. *Front. Plant Sci.*, **8**.

5. Huang, W. and Wilks, A. (2017) A rapid seamless method for gene knockout in Pseudomonas aeruginosa. *BMC Microbiology*, **17**, 199.

6. Taillefer, B., Grandjean, M., Herrou, J., Robert, D., Mignot, T., Sebban-Kreuzer, C. and Cascales, E. (2023) Qualitative and Quantitative Methods to Measure Antibacterial Activity Resulting from Bacterial Competition. *BIO-PROTOCOL*, **13**.

7. Garin, T., Brin, C., Préveaux, A., Brault, A., Briand, M., Simonin, M., Barret, M., Journet, L. and Sarniguet, A. (2024) The type VI secretion system of Stenotrophomonas rhizophila CFBP13503 limits the transmission of Xanthomonas campestris pv. campestris 8004 from radish seeds to seedlings. *Molecular Plant Pathology*, **25**, e13412.

8. Jones, P., Binns, D., Chang, H.-Y., Fraser, M., Li, W., McAnulla, C., McWilliam, H., Maslen, J., Mitchell, A., Nuka, G., *et al.* (2014) InterProScan 5: genome-scale protein function classification. *Bioinformatics*, **30**, 1236–1240.

9. Blum, M., Andreeva, A., Florentino, L.C., Chuguransky, S.R., Grego, T., Hobbs, E., Pinto, B.L., Orr, A., Paysan-Lafosse, T., Ponamareva, I., *et al.* (2025) InterPro: the protein sequence classification resource in 2025. *Nucleic Acids Res*, **53**, D444–D456.

10. Almagro Armenteros, J.J., Tsirigos, K.D., Sønderby, C.K., Petersen, T.N., Winther, O., Brunak, S., von Heijne, G. and Nielsen, H. (2019) SignalP 5.0 improves signal peptide predictions using deep neural networks. *Nat Biotechnol*, **37**, 420–423.

11. Möller, S., Croning, M.D.R. and Apweiler, R. (2001) Evaluation of methods for the prediction of membrane spanning regions. *Bioinformatics*, **17**, 646–653.

12. Abramson, J., Adler, J., Dunger, J., Evans, R., Green, T., Pritzel, A., Ronneberger, O., Willmore, L., Ballard, A.J., Bambrick, J., *et al.* (2024) Accurate structure prediction of biomolecular interactions with AlphaFold 3. *Nature*, **630**, 493–500.

13. Danov, A., Pollin, I., Moon, E., Ho, M., Wilson, B.A., Papathanos, P.A., Kaplan, T. and Levy, A. (2024) Identification of novel toxins associated with the extracellular contractile injection system using machine learning. *Molecular Systems Biology*, **20**, 859–879.

14. Geller, A.M., Shalom, M., Zlotkin, D., Blum, N. and Levy, A. (2024) Identification of type VI secretion system effector-immunity pairs using structural bioinformatics. *Molecular Systems Biology*, **20**, 702–718.

15. Holm, L. (2022) Dali server: structural unification of protein families. *Nucleic Acids Res*, **50**, W210–W215.

16. van Kempen, M., Kim, S.S., Tumescheit, C., Mirdita, M., Lee, J., Gilchrist, C.L.M., Söding, J. and Steinegger, M. (2024) Fast and accurate protein structure search with Foldseek. *Nat Biotechnol*, **42**, 243–246.

17. Pettersen, E.F., Goddard, T.D., Huang, C.C., Meng, E.C., Couch, G.S., Croll, T.I., Morris, J.H. and Ferrin, T.E. (2021) UCSF ChimeraX: Structure visualization for researchers, educators, and developers. *Protein Science*, **30**, 70–82.
