# Supplementary material for: Specific and broad-spectrum antibacterial effectors of type VI secretion system drive competition of Stenotrophomonas rhizophila against bacteria from seed microbiota
Source: Microbiol Spectr. 2026 Jun 15;14(7):e03532-25. doi: 10.1128/spectrum.03532-25 (PMC13340143; doi:10.1128/spectrum.03532-25)
Supplement: Supplemental legends — Legends for supplemental figures and tables. [file spectrum.03532-25-s0003.docx]

**LEGENDS FOR SUPPLEMENTAL FIGURES AND TABLES**

**FIG S1**. **Structural comparison of *S. rhizophila* CFBP13503 VgrG trimers.** Structures of the seven T6SS-associated VgrG trimers were generated with AlphaFold3. Quality scores of structural (pTM) and interaction (ipTM) modelling are indicated. The trimers are coloured from N-terminal (blue) to C-terminal (red) and aligned together with ChimeraX. Accessory C-terminal DUF2345 domains are indicated when present. The length of the VgrG trimer has been measured using AlphaFold3 ruler from the lowest residue to the highest.

**FIG S2. Expression and protein production of VgrG and associated effectors in *S. rhizophila* CFBP13503. A)** Promoter activity of T6SS effector-associated clusters. Fluorescence level of P*vgrG* or P*paar-tse* transcriptional reporter was measured with a spectrophotometer at 570 nm for mCherry detection on exponential or stationary phase cultures. P*tssJ*-*mCherry* was used as a positive control of T6SS expression. A strain containing the promoter-less plasmid pME-*mCherry* was used to normalize the fluorescence (R.F.U). Values combine three independent biological replicates and standard deviation is shown. **B)** RT-PCR of *vgrG* and *paar-tse* genes. mRNA from *S. rhizophila* CFBP13503 WT grown in TSB10 until stationary phase were subjected to reverse transcription into cDNA and PCR amplification for 35 cycles using primers for *vgrG* and *paar-tse* mRNA detection. *tssB* (B), *rrnA* (16S) and *recA* genes were used as positive control for mRNA detection. gDNA was used as a positive control for primer annealing, and raw mRNAs were used as a negative control for DNA contamination. **C)** Promoter activity of T6E-associated clusters under NaCl 1% condition. *S. rhizophila* CFBP13503 strain was grown exponentially in TSB10 (Mock) or in TSB10 supplemented with NaCl 1%. Data came from three independent biological replicates. **D)** Detection of T6SS effectors and VgrG proteins in the secretome of stationary growth phase *S. rhizophila* cells. Spectral counts of each protein detected in *S. rhizophila* WT supernatant were subtracted to that of Δ*hcp* strain to observe the T6SS-associated secretome. Data came from four biological replicates, error bars representing the spectral count variability.

**FIG S3. *S. rhizophila* CFBP13503 T6SS dynamic and model of activity** **A)** Qualitative competition assay (LAGA) against *E. coli* as a target and *S. rhizophila* CFBP13503 WT or derivatives as attackers. Colour from yellow to purple is indicative of the increasing cell lysis (Taillefer et *al*., 2023). Δ*tssB* strain was used as a negative control of T6SS antibacterial activity (Garin et *al*., 2024). *E. coli* alone (Ec -) or *S. rhizophila* WT (*Sr* -) alone indicate no spontaneous lysis or coloration. B-GFP strain shows similar antibiosis activity than the WT. **B)** Frequency and cumulative frequency of T6SS+ cell in stationary growth phase during 70 min time-lapse. The time-lapse consisted in the acquisition of 15 frames of 5 min intervals with a GFP filter and resulted in the measurement of 940 stationary phase cells over 4 different fields. This measure shows that every image presents the same T6SS+ cell frequency, while over 80% of the population has been counted to fire at least one time during the experiment (cumulative frequency: sum of the current and the previous time frame frequencies). **C)** Close-up of the T6SS dynamic in a *S. rhizophila* B-GFP cell with 20 second interval resolution. Images show the highly dynamic T6SS with a cycle from assembly to firing of approximately 60 seconds and a refractory period of 60-80 seconds (left). Model of the polar, short, highly dynamic *S. rhizophila* CFBP13503 T6SS (right).

**FIG S4**. **Susceptibility profile of 30 selected seed-associated bacteria to *Sr*-T6SS.** Competition assay between seed-associated bacteria and *S. rhizophila* CFBP13503. Survival is measured by CFU counting or Survivor Growth Kinetic (SGK) after 6h co-culture on TSA10 (for gram-negative targets) or TSA100 (for gram-positive targets) with *S. rhizophila* WT or Δ*hcp* strains as attackers. Represented mortality is the log_10_ change observed between WT and Δ*hcp* conditions. Phylogenetic distance is indicated as described by Garin et *al*. (2025). The most phylogenetically distant bacteria from *Rhizobiaceae* and *Microbacteriaceae* are the least susceptible to *Sr*-T6SS (i.e. resistant strains, < 1 log mortality, orange bars). Target strain identity is coded as described in Table S1.

**FIG S5**. **Distribution of *S. rhizophila* T6SS effector (*Sr*-T6Es) proteins and *immunity (Sr*-T6Is) proteins in 177 seed-associated bacteria**. Pie charts display the frequency of *Sr*-T6E (left) and *Sr*-T6Is (right) distribution as the presence of at least one over all 177 strains that possess it (*Sr*-T6E, n=44; *Sr*-T6I, n=33). Homologues V1-V7, P1-P5, Tli1-Tli7 and Tdi1-Tdi5 were counted as the same *Sr*-T6E or *Sr*-T6I.

**FIG S6. Quantitative competition assay between *S. rhizophila* CFBP13503 WT or T6SS effector (T6E) deletion mutants and seed-associated bacteria.** Barplot indicates the target strain mortality when confronted to *S. rhizophila* WT or the T6E deletion mutants compared to the T6SS-deficient Δ*hcp* strain. Mortality was measured by SGK method or CFU counting (indicated by “C” in the graph title). Values combine three or four biological replicates and error bars show standard deviation. Differences between WT and T6E deletion mutants were assessed using a Wilcoxon test (*P* < 0.05: *).

**FIG S7. Effect of *S. rhizophila* T6SS effector(T6E) deletion mutants on cell lysis phenotype.** Qualitative competition assay (LAGA) between *E. coli* W3110 (top) or *Xcc* 8004 *lacZ*+ (bottom) and *S. rhizophila* WT or T6E deletion mutants grown in stationary growth phase. Target cell lysis was revealed by addition of CPRG after 2h of coincubation on TSA10.

**FIG S8. Seed-associated bacteria aggressive status and their impact on *paar5-tde5* promoter induction A)** Aggressiveness of strains toward *E. coli* W3110. Qualitative competition assay (LAGA) between *E. coli* and selected seed-associated bacteria. A co-culture was performed by mixing T6SS+ seed-associated bacteria (Sr, Pa1 and Ox2) as attacker and *E. coli* W3110 (Ec) as a target on TSA10 agar plate for 6h. Target cell lysis was revealed by addition of CPRG. A yellow color indicates no target cell lysis, and a purple color indicates a high level of lysis. Intermediate colors indicate a low to medium level of target cell lysis. *S. rhizophila* WT (Sr) was used as a positive control of lysis-associated antibacterial activity. *E. coli* and attackers alone (*Ø = no Ec*)) were used as a negative control that showed no spontaneous CPRG hydrolysis. *Sr*: *S. rhizophila* CFBP13503; *Pa*1: *P. agglomerans* 1 CFBP13505; *Ox*2: *Oxalobacteraceae* 2 CFBP8753. **B)** P*paar5-tde5* activity during a competition assay. A co-culture on TSA10 was performed in a 96-well microplate by mixing CFBP13503 pME-P*paar5-tde5::*mCherry with Pa1, Ox2, Ec and Sr CFBP13503 WT (Sr) or Δ*hcp*. The two last conditions are controls of P*paar5-tde5* activity when alone. A negative control of fluorescence was achieved by mixing Sr CFBP13503 WT and Δ*hcp* (Sr+Δhcp). Co-cultures were incubated 24h at 28°C in a microplate reader where OD_600_ and F_570_ were acquired every 20 minutes. Curves combine ten biological replicates and error bars are indicated as standard deviation. Means of Sr condition and Pa1, Ox2 or Ec conditions at 10, 15, 20 and 25 hours were compared with a Wilcoxon test. Statistical results between Sr and Ox2 or Ec is indicated (p-value > 0.05: ns; p-value < 0.0001: ***).

**FIG S9. Impact of V5V2b deletion on target cell death phenotype. A)** Representative images of microscopy time-lapse of a competition assay between *E. coli* W3110 mCherry and *S. rhizophila* WT or V5V2b double deletion mutant. Stationary growth phase cells of the attacker and the target were mixed and spotted onto an agar pad for microscopy imaging of the competition. Time-lapse was performed over 1h and imaging with a 5 min interval using an mCherry filter for target strain detection. Colored arrows indicate the cell death event in the next image. **B)** Statistical analysis of cell death event occurring during a competition between *E. coli* W3110 mCherry and *S. rhizophila* CFBP13503 WT or V5V2b double deletion mutant under the microscope. Each target cell death is manually counted as an event. Values combine cell death events over three different microscopy fields.

**FIG S10. Quantitative competition assay between *S. rhizophila* CFBP13503 WT or putative amidase deletion mutants and seed-associated bacteria.** Barplot indicates the target strain mortality when confronted to *S. rhizophila* WT or the double deletion mutants compared to the T6SS-deficient Δ*hcp* strain. Mortality was measured by SGK method or CFU counting (indicated by “C” in the graph title). Values combine three or four biological replicates and standard deviation is shown. Differences between WT and T6SS effector double deletion mutants were assessed using a Wilcoxon test (*, *P* < 0.05).

**Table S1**. **Genomic composition of *vgrG* and *paar-tse* clusters of *S. rhizophila*.**

**Table S2**. **List of strains used in this study.**

**Table S3. List of primers used in this study.**

GC content is coloured from lowest (blue) to intermediate (white) highest (red). Predicted function was done using several predictive tools and structure comparison such as InterProScan, HMMER, FoldSeek and DALI server.

**Table S4**. **Matrix of prediction of interactions between one T6SS effector (T6E: V1-7 or P1-4) and the proteins encoded in the corresponding *vgrG* or *paar* cluster.**

AlphaFold3 ipTM score is indicated and coloured from lowest (white) to highest (red). A confident interaction is predicted by an ipTM score > 0.75. When ipTM < 0.75, the highest score was considered as a positive interaction (V7). The cognate immunity is indicated with a bold score. Analysed in August 2025.

**Table S5**. **Presence of** ***S. rhizophila* CFBP13503 T6SS immunity (*Sr*-T6I) protein orthologues in 177 seed-associated bacteria and the predicted inhibition of the cognate *S. rhizophila* CFBP13503 effector.**

AlphaFold3 ipTM score is indicated and coloured from lowest (white) to highest (red). A confident protein-protein interaction is predicted by an ipTM score > 0.75. The *Sr-T6E-I* pair from the CFBP13503 strain is used as the reference for a positive interaction (CFBP13503 line). Strains selected for competition assays are indicated in a grey shaded cell. Empty cells indicate the absence of orthologous immunity.

**Table S6**. **Presence of *S. rhizophila* CFBP13503 T6SS effector (T6E) protein orthologues in 177 seed-associated bacteria**.

The number of orthologues in the same strains is indicated. The bold number in the column V1-V7 indicates that the effector is more similar to V7. Strains selected for competition assays are indicated in a grey shaded cell. Empty cells indicate the absence of an orthologous effector.

**Table S7. Distribution of *S. rhizophila* CFBP13503 T6Es homologues among bacterial diversity.**

Each genus reported are top 100 hits from a NCBI BlastP search, thus representing the closest amino-acid sequence (august 2025).

**Table S8. List of proteins detected in total cell proteome and culture supernatant of *S. rhizophila* CFBP13503.**

WS_S: stationary CFBP13503 WT supernatant; HS_S: stationary CFBP13503 Δ*hcp* supernatant; W_S: stationary CFBP13503 cell pellet.

**Table S9. List of accessions of the seed-borne bacterial genomes from the CFBP collection.**
