# Supplementary material for: Specific and broad-spectrum antibacterial effectors of type VI secretion system drive competition of Stenotrophomonas rhizophila against bacteria from seed microbiota
Source: Microbiol Spectr. 2026 Jun 15;14(7):e03532-25. doi: 10.1128/spectrum.03532-25 (PMC13340143; doi:10.1128/spectrum.03532-25)
Supplement: Supplemental file — Time lapse of killing phenotypes of Sr against Xcc and Ec [file spectrum.03532-25-s0005.pptx]

## Slide 1
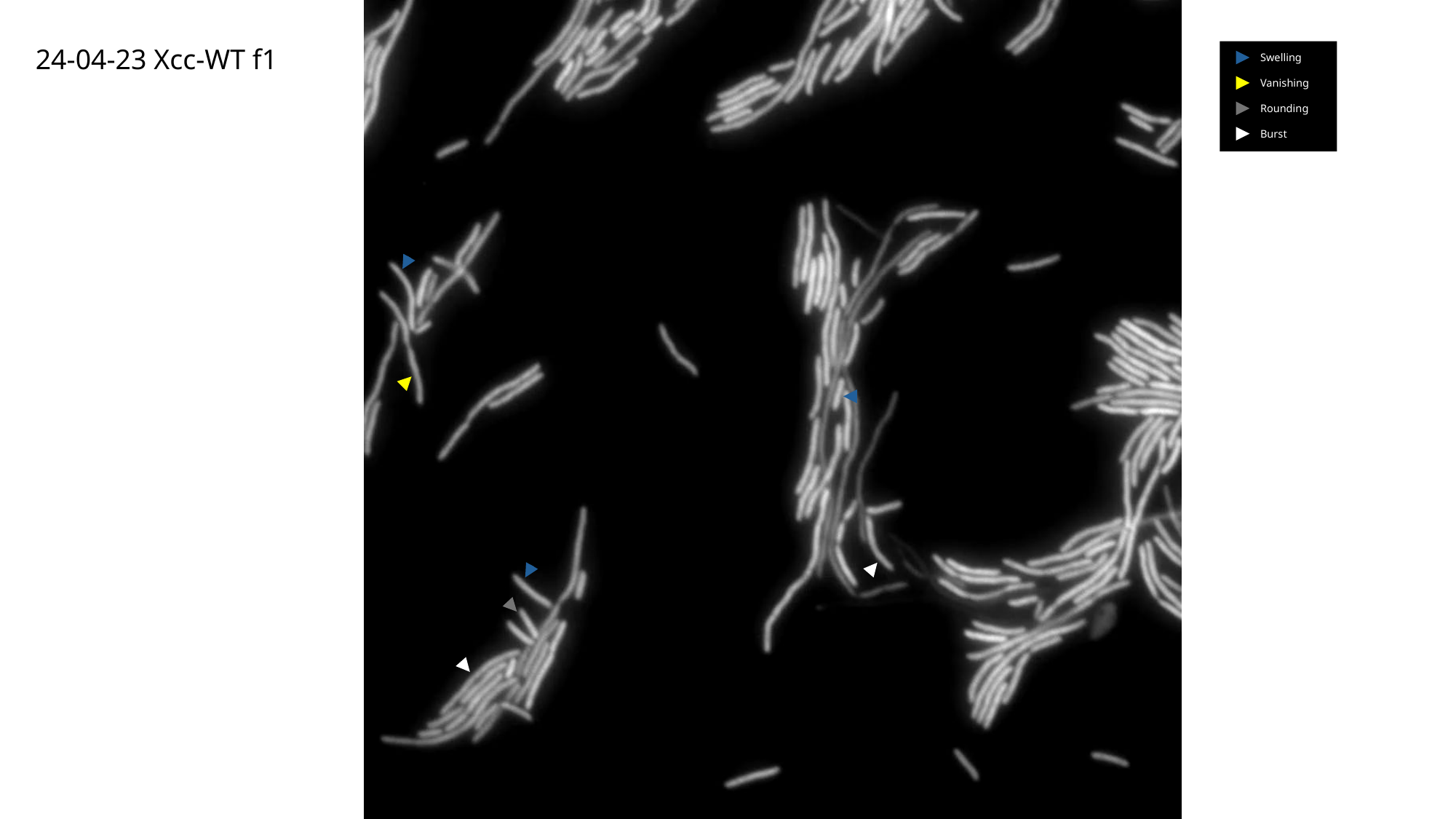

24-04-23 Xcc-WT f1
Swelling
Vanishing
Rounding
Burst

## Slide 2
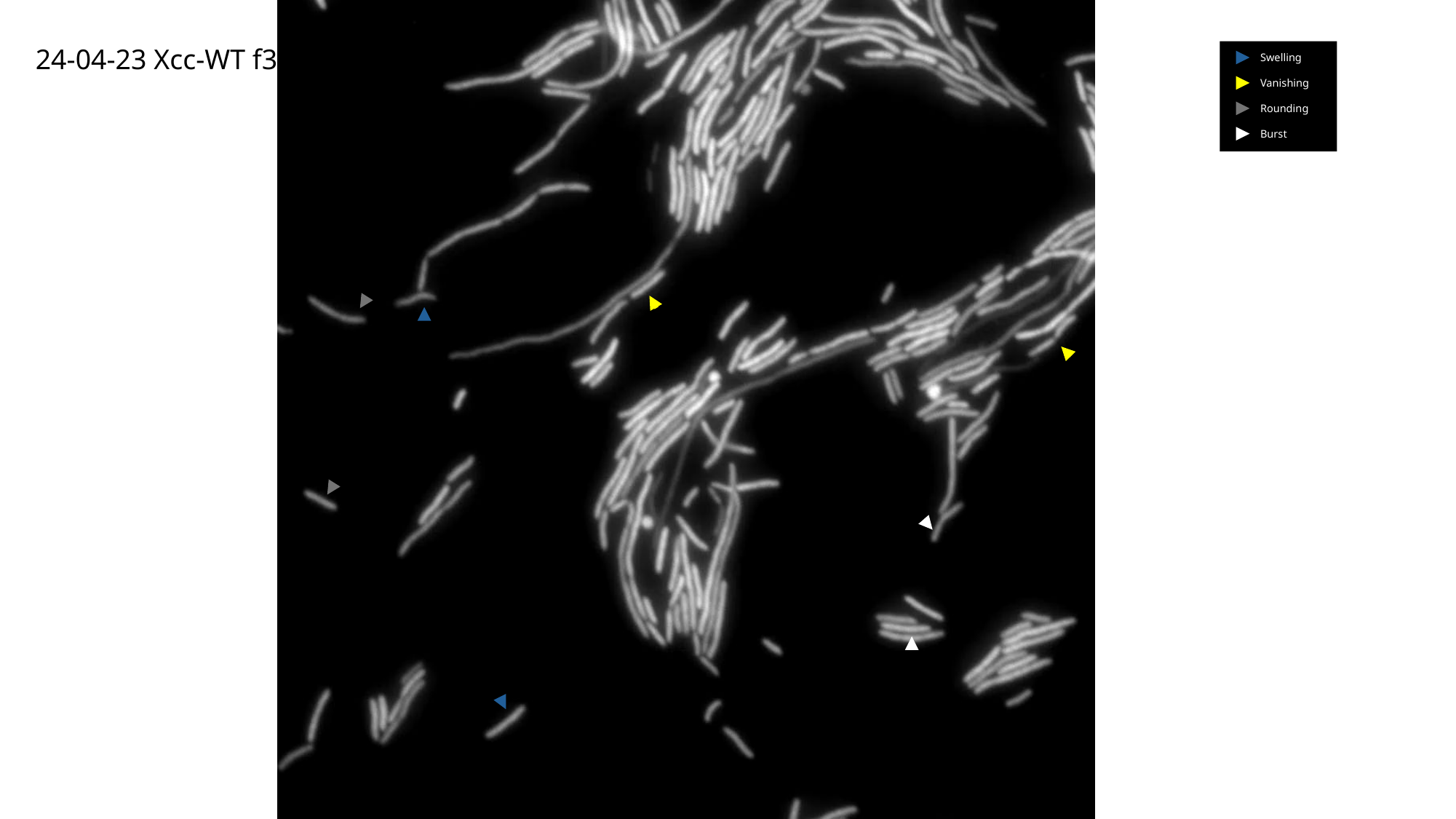

24-04-23 Xcc-WT f3
Swelling
Vanishing
Rounding
Burst

## Slide 3
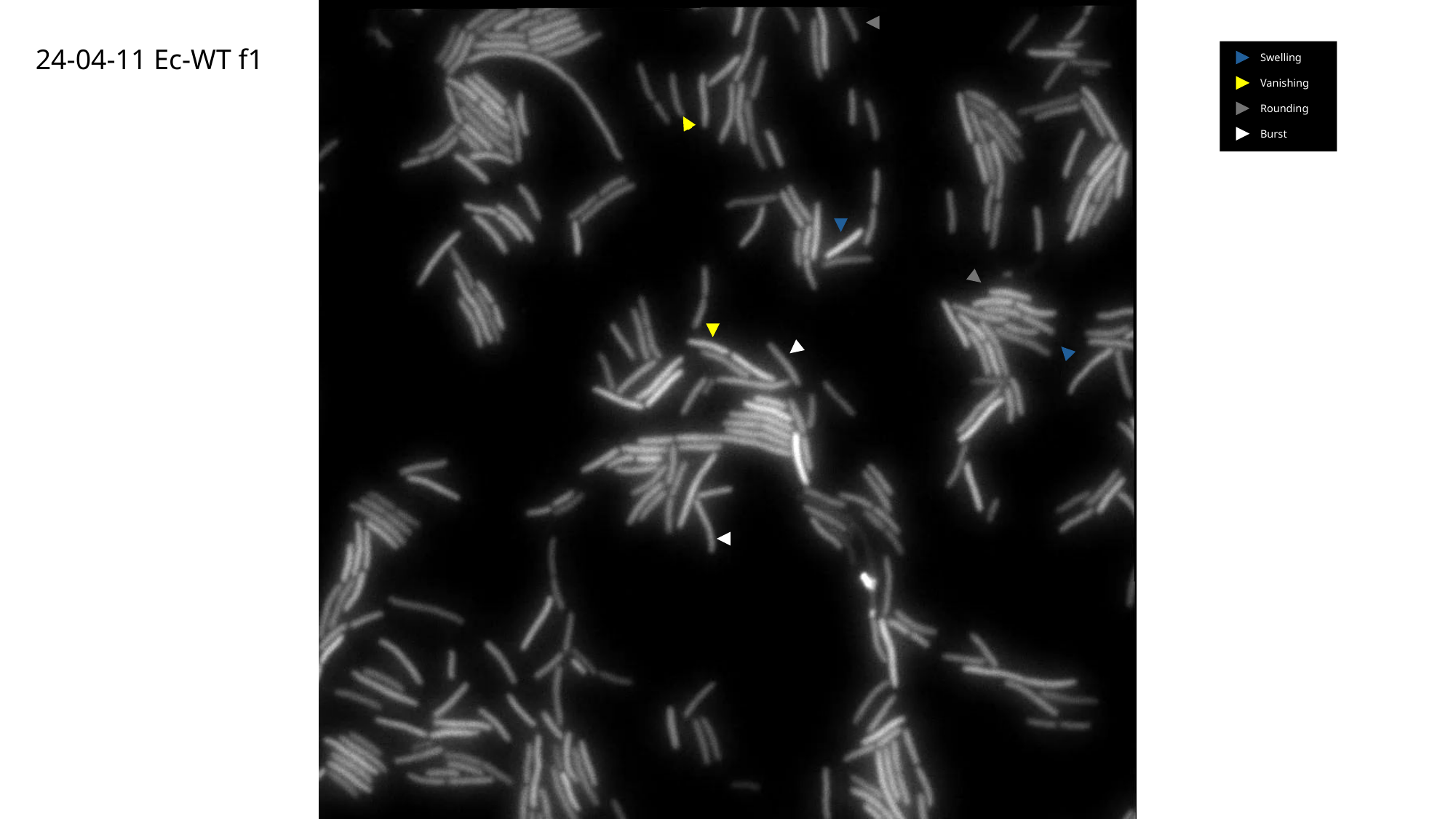

24-04-11 Ec-WT f1
Swelling
Vanishing
Rounding
Burst
